# Assessment of Polysaccharides from Mycelia of genus *Ganoderma* by Mid-Infrared and Near-Infrared Spectroscopy

**DOI:** 10.1038/s41598-017-18422-7

**Published:** 2018-01-08

**Authors:** Yuhan Ma, Huaqi He, Jingzhu Wu, Chunyang Wang, Kuanglin Chao, Qing Huang

**Affiliations:** 1 Key Laboratory of High Magnetic Field and Ion Beam Physical Biology, Institute of Technical Biology and Agriculture Engineering, Hefei Institutes of Physical Science, Chinese Academy of Sciences, Hefei, 230031 China; 2 National Synchrotron Radiation Laboratory (NSRL), School of Life Science, University of Science and Technology of China (USTC), Hefei, 230026 China; 3 College of Life Science, Anhui Science and Technology University, Fengyang, 233100 China; 4 School of Computer and Information Engineering, Beijing Technology and Business University, Beijing, 100048 China; 5 Environmental Microbial and Food Safety Laboratory, Agricultural Research Service, USDA, Beltsville, MD 20705 USA

## Abstract

*Ganoderma lingzhi* (*G. lingzhi*), *G. sinense*, *G. applanatum*, etc. belongs to the *Ganoderma* genus of polypore mushrooms which contain rich polysaccharides valuable for nutrition and positive medicinal effects. In order to evaluate polysaccharide content in *Ganoderma* mycelia obtained in the fermentation process quickly and accurately, in this work we employed infrared spectroscopy to examine different *Ganoderma* stains of samples from diversified sources. Through mid-infrared (mid-IR) spectroscopy, we could identify the most relevant spectral bands required for polysaccharide evaluation, and through near-infrared (NIR) spectroscopy, we could establish the quantification model for making satisfactory prediction of polysaccharide ingredient content. As such, we have achieved an effective and convenient approach to quantitative assessment of the total polysaccharides in *Ganoderma* mycelia but also demonstrated that infrared spectroscopy can be a powerful tool for quality control of *Ganoderma* polysaccharides obtained from industrial production.

## Introduction

*G. lingzhi*, *G. sinense*, *G. applanatum*, etc. belongs to the genus *Ganoderma* which has been adopted as traditional Chinese herbal medicine in China since ancient times^1,2^. Nowadays, researchers have proved that *G. lingzhi* and other *Ganoderma* species such as *G. sinense*, *G. applanatum*, etc. possess encouraging medical curing effects on hepatopathy, chronic hepatitis, nephritis, hypertension, arthritis, neurasthenia, insomnia, bronchitis, asthma, and gastric ulcers^3,4^.

The major pharmacological ingredients of mushrooms in the *Ganoderma* genus include Ganoderma polysaccharides and ganoderic acids, and in particular, it has been reported that Ganoderma polysaccharides have some special functions such as therapeutic effects on cancer^5–7^, obesity^8,9^, diabetes^10,11^, and pancreatitis^12^; as well as pharmaceutical activity on immune modulation^13–17^, liver protection^4,18,19^, and vascular endothelial cell growth inhibition^20,21^. Especially, it has been reported that β-glucans in *Ganoderma* are closely related to health, for they can stimulate the immune response through cytotoxic or immunomodulatory mechanisms to enhance cellular immunity, and stimulate a variety of immune cells including macrophages^22–24^, neutrophils^25,26^, natural killer cells^27,28^ and dendritic cells^29,30^. In fact, *Ganoderma* polysaccharides extracted from mycelia and spores are used as prescription drugs in China by many pharmaceutical factories and companies.

Conventionally, polysaccharides can be examined through biochemical approaches such as the phenol–sulphuric acid method or anthrone–sulphuric acid method^31^. However, these methods normally require deleterious substances such as phenol, anthrone, and sulfuric acid^32^. In order to achieve a more convenient approach to quantitative assessment of polysaccharides in *Ganoderma mycelia*, non-destructive spectroscopic approaches including infrared spectroscopy thus become especially attractive for their outstanding advantages in cost, efficiency, sample preparation and instrument operation. In fact, infrared spectroscopy (including both mid-infrared and near-infrared spectroscopy) is widely used today in the agriculture^33,34^ and pharmaceutical industry^35,36^, and shows great potential for the application in food processing industry as well^37,38^.

In this context, therefore, we intended to employ infrared spectroscopy to assess polysaccharides in mycelia of different *Ganoderma* strains produced in the fermentation process. Although some previous studies have reported the application of both mid-IR- and NIR- spectroscopy in analysis of *Ganoderma* polysaccharides^31,39^, there are still some problems yet to be solved for practical applications. For example, in the industrial production of *Ganoderma* polysaccharides, it is standard to produce *Ganoderma* mycelia through liquid fermentation, but there is neither a report for analysis of *Ganoderma* mycelia through mid-infrared (mid-IR) spectroscopy, nor a report of suitable near-infrared (NIR) quantification, that is valid for the assessment of polysaccharides in *Ganoderma* mycelia produced during the fermentation process. Therefore, in this work, we intended to combine both mid-IR and NIR techniques so that we could not only qualitatively analyze the samples based on mid-IR spectral data, but also quantitatively evaluate the polysaccharides in *Ganoderma* mycelia based on the optimized NIR quantification model.

## Results

### Analysis of Ganoderma mycelia via mid-IR spectroscopy

**Figure 1:**
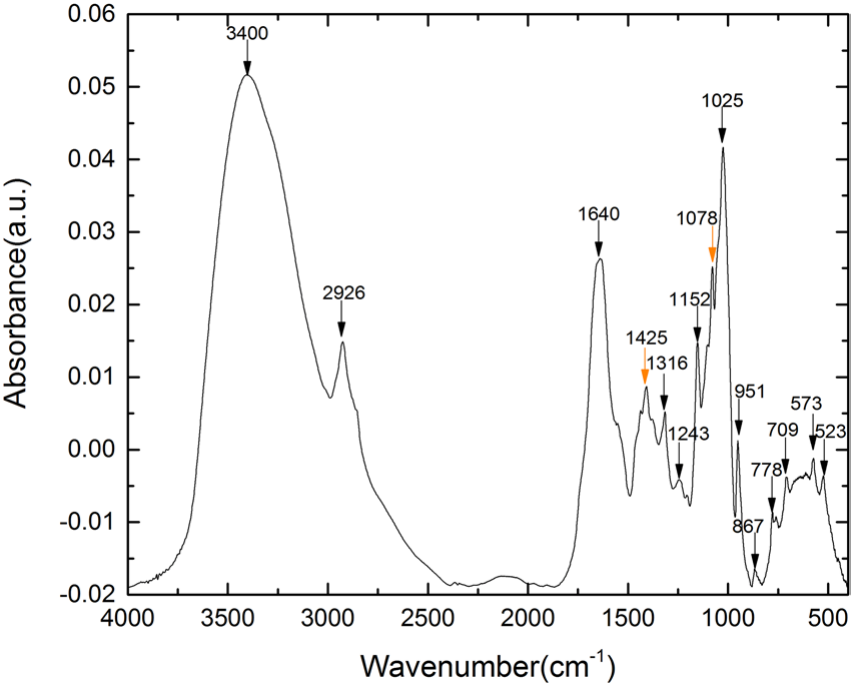
The mid-IR spectra of *G. lingzhi* mycelium dried powder.

Mid-IR and NIR are both forms of electromagnetic radiation with wavelengths longer than visible light. The wavelength of mid-IR is between 4000–400 cm^−1^ (2.5–25 μm), while the wavelength of NIR with is between 14,000–4000 cm^−1^ (0.8–2.5 μm)^40^. The absorption of mid-IR involves transitions between vibrational energy states and rotational sub-states of the molecule which can thus be employed for the elucidation molecular structure^40,41^. A typical mid-IR spectrum of *G. lingzhi* mycelia is showed in Fig. 1, where the characteristic bands are shown with their assignments given in Table 1. The bands assigned to carbohydrates include the following: the bands at 1425 cm^−1^ ^42^, 1316 cm^−1^ ^43^, 1152 cm^−1^ ^44^, 1078 cm^−1^ ^45^, 1025 cm^−1^ ^46,47^, and 951 cm^−1^ ^47,48^.

**Table 1:** Assignments of the characteristic mid-IR bands in the mid-IR spectrum of *G. lingzhi mycelium*.

Wavenumber(cm^−1^)	Functional Group Assignments
3400	-OH stretching^55^
2926	CH_2_ asymmetric stretching^56^
1640	Amide I^56^
1457	CH_2_ in polysaccharides^57^
1425	C-H deformation in lignin and carbohydrates^15^
1372	C-H in-plane bending vibration^58,59^
1314–1316	symmetric CH_2_ bending of cellulose^43^
1243	COH in-plane bending/CH in-plane bending^58^
1152–1156	C-O-C asymmetric stretching of glycosidic linkage^44^
1078	C-O stretching of β-glucans^45,46,51^
1044	stretching vibration of C-O-C group^60^
1025	stretching vibration of C-O α-glycosidic bond^47^
951	β-glycosidic bond^47^; C-O and C-C stretching^48^
867	γ (C-H)^61^; furanose ring^62^
778	COO^−^ deformation^63^
709	CH out-of plane bending^64^
573	bending vibration of a polysaccharide ring^39^
523	pyranose ring^62^; C=O asymmetric deformation^65^

**Figure 2:**
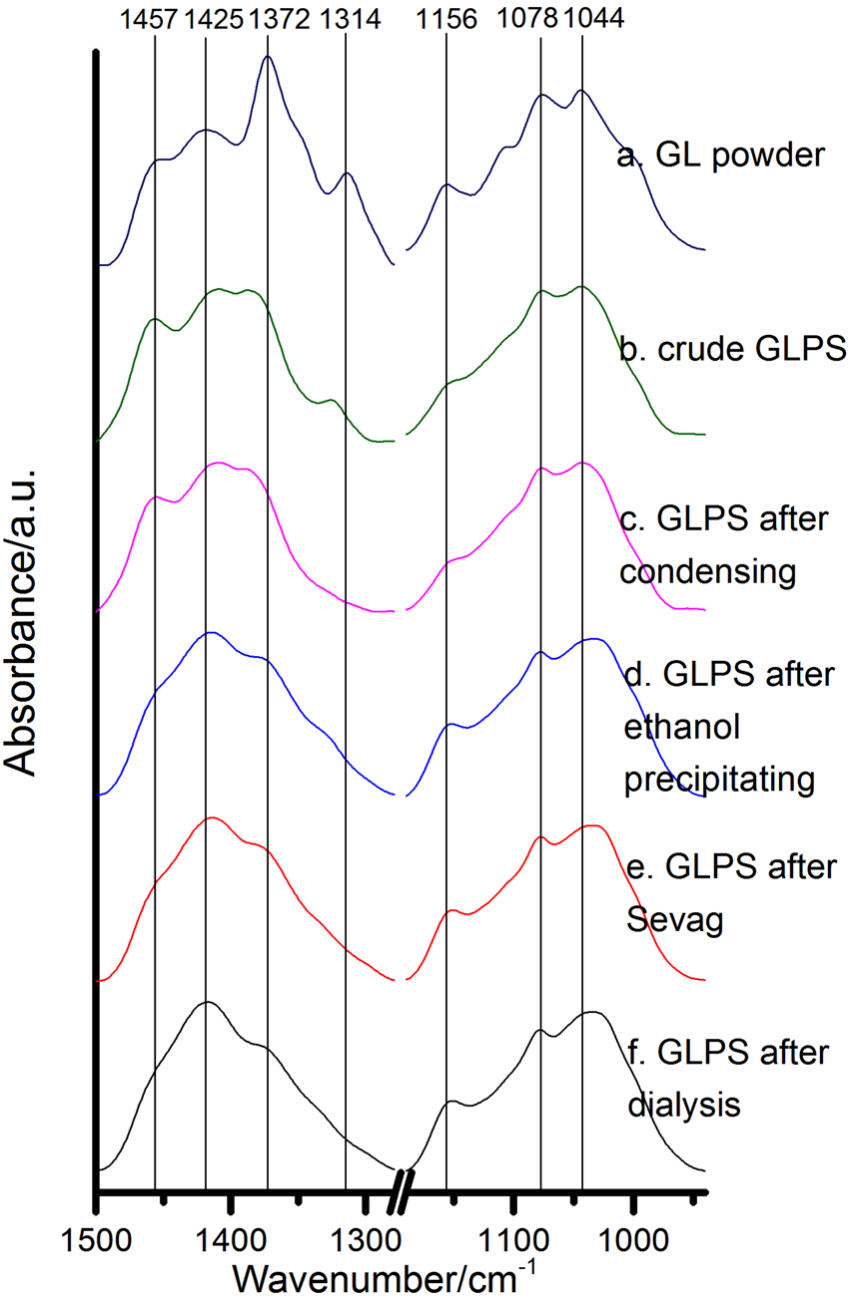
The mid-IR spectra of different samples obtained from the *G. lingzhi* polysaccharide extraction process. (**a**) GL powder refers to the drying powder from wet *G. lingzhi* culture mycelia; (**b**) crude GLPS refers to the extracted crude polysaccharide sample from the dried powder in 70 °C of hot water; (**c**) GLPS after condensing refers to the condensing supernatant sample using rotary vacuum approach; (**d**) GLPS after ethanol precipitating refers to the condensed remnant sample after ethanol precipitating process; (**e**) GLPS after Sevag refers to the sample with proteins removed with the by Sevag method; and (**f**)GLPS after dialysis refers to the sample which removed small molecular impurity substances after dialyzing process.

In addition, we also measured and analyzed different *Ganoderma* samples obtained in different stages during polysaccharide extraction process, with their mid-IR spectra shown in Fig. 2. From the spectrum, we can identify the characteristic bands attributed to carbohydrates, and we notice especially that the relative intensities of the bands at 1425 cm^−1^ and 1078 cm^−1^ are stronger for the samples with high content of polysaccharide after the extraction procedure.

### Assessment of polysaccharide in Ganoderma mycelia by NIR spectroscopy

**Figure 3:**
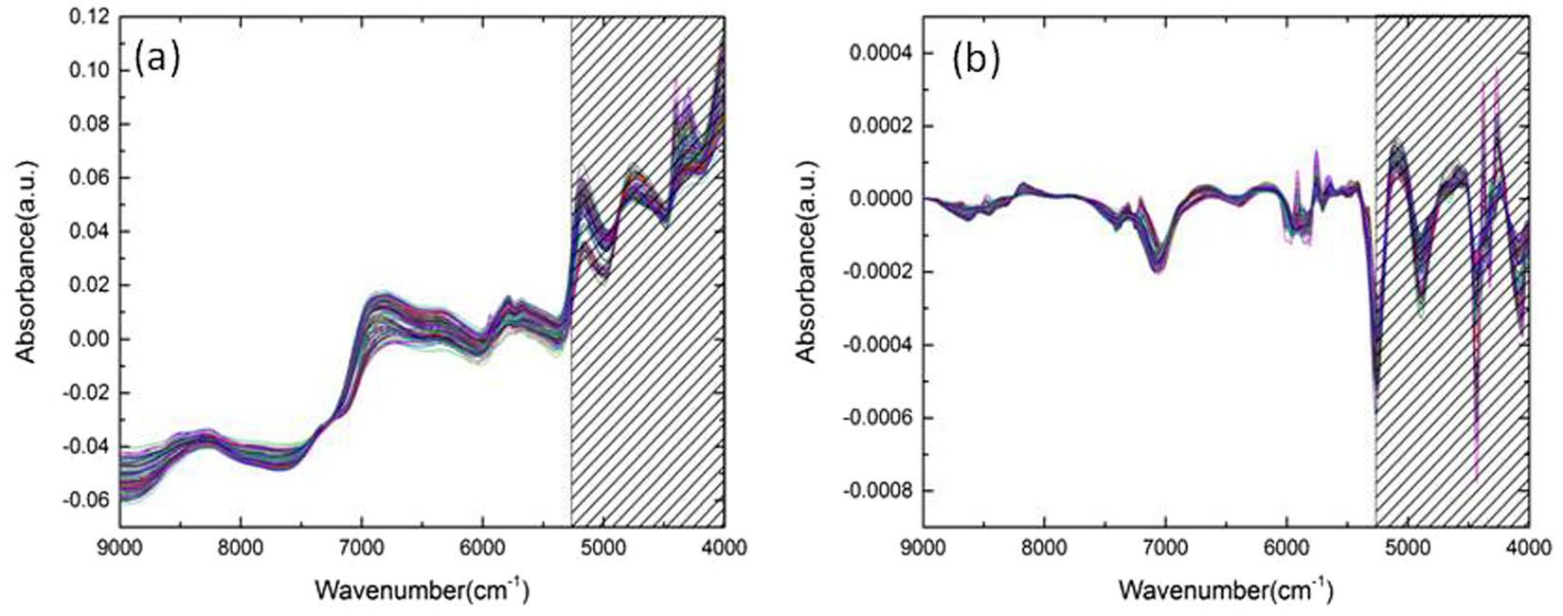
NIR spectra of *Ganoderma* mycelia with showing the selection of spectral range of 5268.8–4000 cm^−1^ for the optimal quantification assessment. (**a**) The conventional NIR spectra of *Ganoderma* mycelia; (**b**) The first derivative spectra of the corresponding NIR spectra.

In addition, we employed the NIR spectroscopy for the quantification of the polysaccharide content in *Ganoderma* mycelia. Generally, NIR spectroscopy measures the broad overtone and combination bands of some of the fundamental vibrations and can be an excellent technique for rapid and quantitative evaluation of many chemicals^40^. Figure 3a shows the NIR spectra in the 9000–4000 cm^−1^ region, and the corresponding first derivative spectra are shown in Fig. 3b. The absorption peaks at 8403 cm^−1^, 6896 cm^−1^, 5155 cm^−1^ are attributed to water^49^, while the bands at 4307 cm^−1^, 4405 cm^−1^, 5787 cm^−1^, and 5935 cm^−1^ are ascribed to carbohydrate. The NIR spectral band assignments are listed in Table 2.

**Table 2:** Assignments of the NIR absorption bands.

Wavenumber (cm^−1^)	Type	Feature(s)
8403	combination of the first overtone of the O–H stretching and the OH-bending band (2ν_1,3_ + ν_2_)	Water^66^
6896	first overtone of the OH-stretching band (2ν_1,3_)	Water^66^
6674	first overtone of the OH-stretching band	alcohol or water^66^
6307	first overtone of the OH-stretching band	alcohol or water^66^
5935	C–H stretching first overtone	Lignin^67^, hemicelluloses^67,68^
5787	C–H stretching (1st overtone) of –CH_2_	Carbohydrates^69^
5155	combination of stretching and deformation of the O-H group in water	Water^70^
4719	O-H and C-O stretching combination	Polysaccharide^71^
4405	O-H stretching and C-O stretching combination	Polysaccharides^72^
4307	C-H stretching and C-H_2_ deformation combination	Polysaccharides^66^
4021	C-H stretching and C-C stretching combination	Cellulose^66^

**Figure 4:**
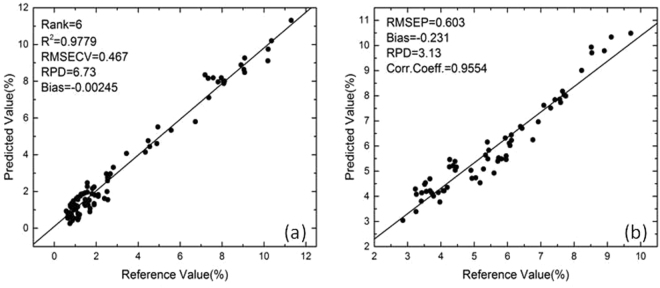
The NIR-based quantitative model for the polysaccharides on the range of (5268.8–4000 cm^−1^) of calibration set (**a**) and prediction set (**b**).

To establish an optimal quantification model for polysaccharide assessment, we employed the methods of moving window partial least-squares (mwPLS) and interval PLS (iPLS) to find the appropriate spectral range for NIR quantification model. The mwPLS analysis (Figure S1a) shows that in the range of (5268.8–4000 cm^−1^) the relative low root mean square error of cross validation (RMSECV) values are relatively low, and the iPLS analysis (Figure S1b) gives rise to the same consistent result. Accordingly, we took this range (5268.8–4000 cm^−1^) and applied the constant offset elimination pre-treatment method for construction of the optimal quantification model. Our result shows that the optimal spectral range for *Ganoderma* mycelia is between 5268.8–4000 cm^−1^, and the pre-treatment method is constant offset elimination (the comparisons using other different pre-treatment methods are given in the supplementary part Table S1). For the calibration set, we achieved determination coefficient (R^2^) = 0.9779, RMSECV = 0.467, RPD = 6.73 at rank = 6, and for the prediction set we obtained root mean square error of prediction (RMSEP) = 0.603, relative percent deviation (RPD) = 3.13, correlation coefficient (corr. coeff.) = 0.9554. The calibration and prediction results are showed in Fig. 4. To check the model efficiency, the plot of RMSECV vs. Rank is also depicted and exhibits a smooth descent line (Figure S2).

## Discussion

### Qualitative analysis of Ganoderma mycelia based on mid-IR and NIR spectroscopy

**Figure 5:**
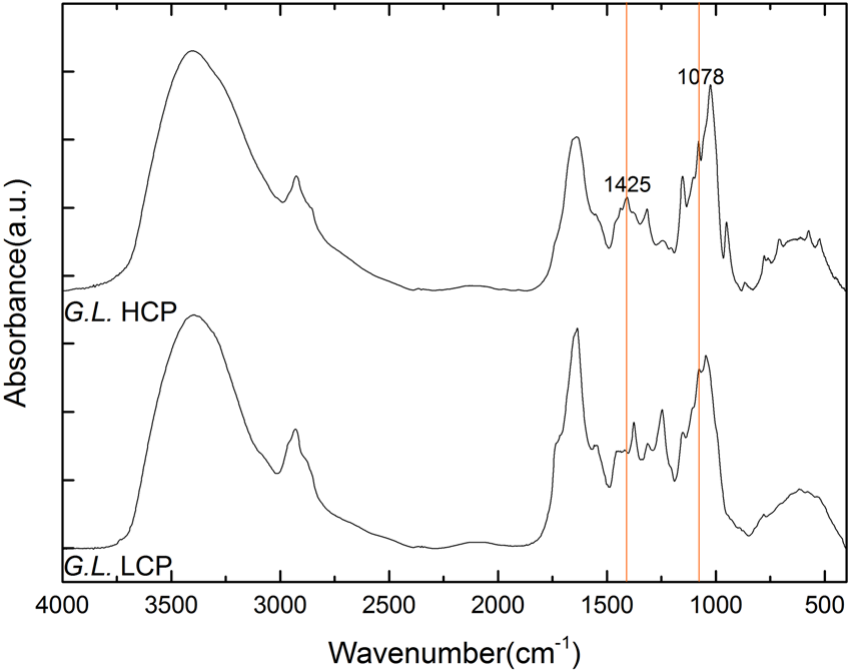
The mid-IR spectra of HCP and LCP *G. lingzhi* mycelia. HCP: high content of polysaccharide; LCP: low content of polysaccharide.

**Figure 6:**
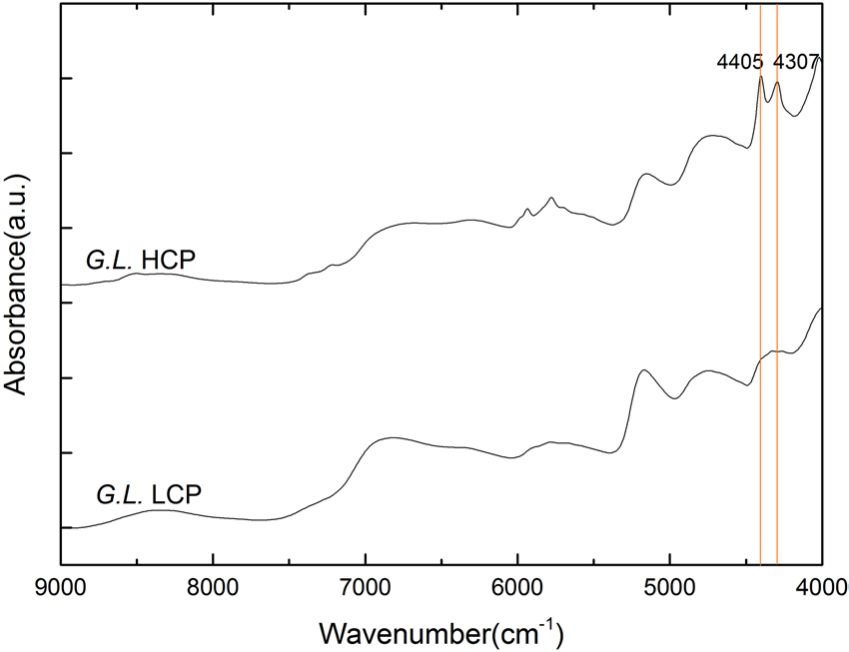
The NIR spectra of both HCP and LCP *G. lingzhi* mycelia, showing the most critical characteristic bands for assessment of polysaccharides.

In the foregoing sections, we have observed and identified the bands at 1425 cm^−1^ and 1078 cm^−1^ which are assigned to *Ganoderma* polysaccharides. These two bands are actually the most distinctive polysaccharide bands for our *Ganoderma* mycelia specimens. To confirm this, we measured the samples of high-content-polysaccharide (HCP) and low-content-polysaccharide (LCP) *G. lingzhi* strains, respectively, with the comparison of their mid-IR and NIR spectra as shown in Figs 5 and 6, respectively. We can see the prominent difference in intensity for the peaks at 1425 cm^−1^ and 1078 cm^−1^, and correspondingly, we can also identify the most relevant polysaccharide bands in NIR spectra at 4307 cm^−1^, 4405 cm^−1^. These mid-IR and NIR bands are actually most useful for polysaccharide detection and quantification.

**Figure 7:**
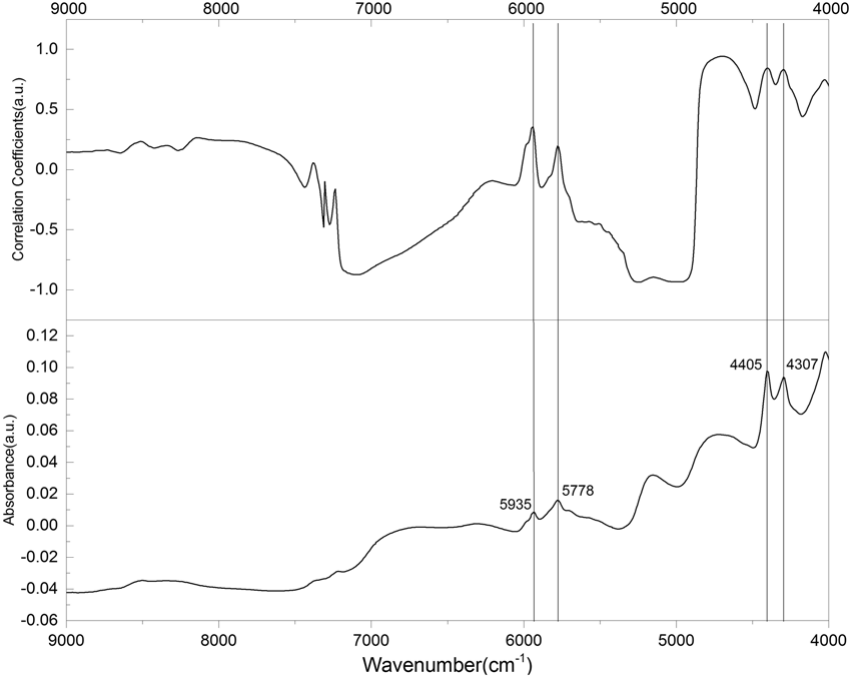
Correlation coefficients of the chemical measurements with the comparison with NIR spectra.

Furthermore, for the NIR analysis, we obtained the correlation coefficient curves based on the NIR spectra, and confirmed that the larger correlation coefficients also occur at these NIR spectral peak positions (Fig. 7).

**Figure 8:**
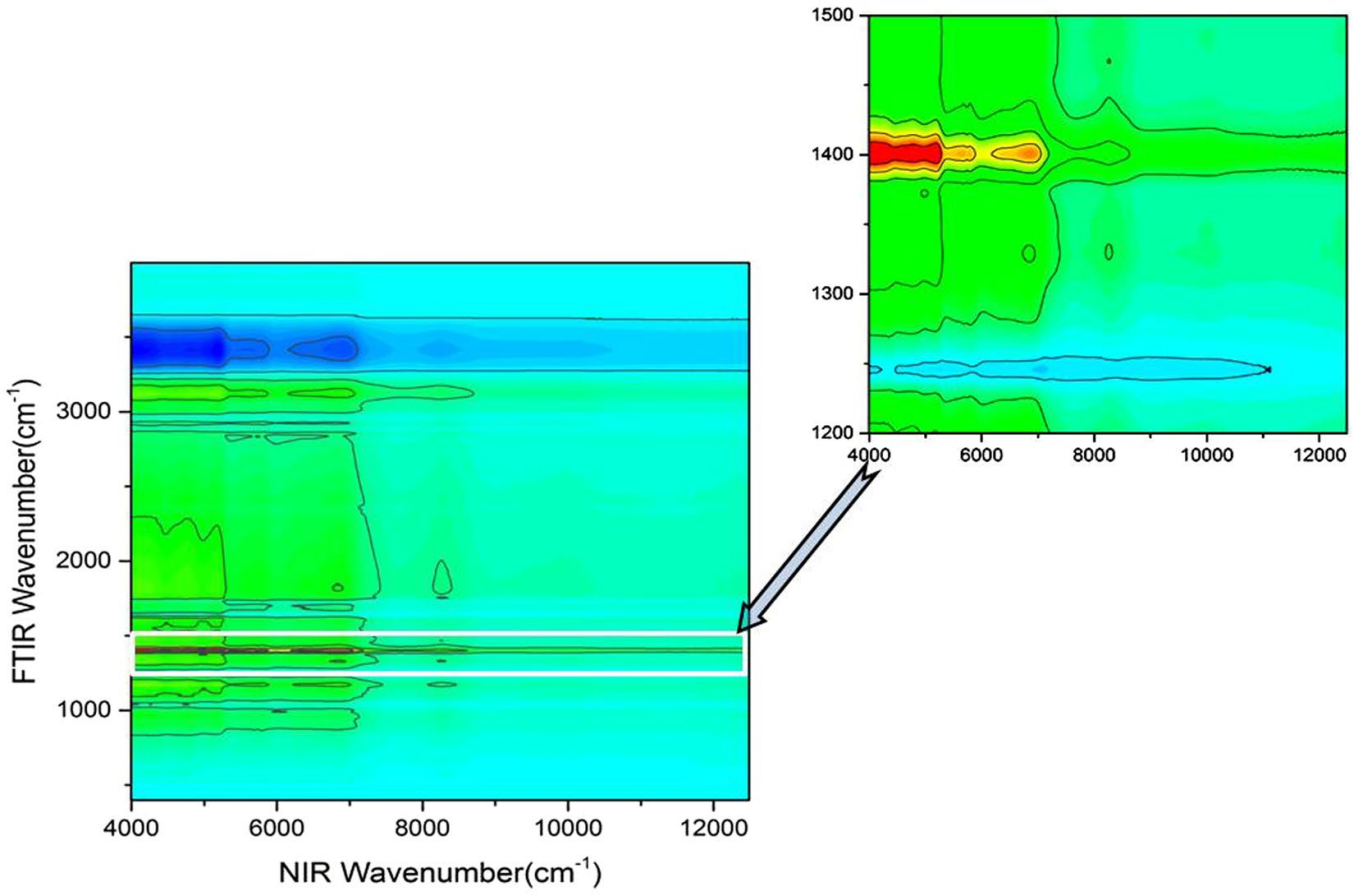
Correlation spectroscopy of NIR and mid-IR spectra of *Ganoderma* mycelia, showing that the NIR spectral range (5268–4000 cm^−1^) is most related to the mid-IR spectral range (1422–1376 cm^−1^).

These peaks are just assigned to polysaccharides (see the NIR band assignments listed in Table 1), and for this reason, our quantification model therefore covers the spectral range of 5268.8–4000 cm^−1^ including the prominent bands at 4307 and 4405 cm^−1^, which are closely related to their mid-IR counterparts, namely, the bands at 1425 cm^−1^ and 1078 cm^−1^. These two mid-IR bands are actually corresponding to the C-H bending and C-O-H bending from pyranose ring of glucan, respectively, which exist widely in different kinds of mushrooms^50^. More specifically, the characteristic mid-IR peak 1078 cm^−1^ is assigned to the C-O stretching in β-glucans of lignin and carbohydrates^51^, which is related with 4405 cm^−1^ in NIR spectra for it stems from O-H stretching and C-O stretching combination. The 1425 cm^−1^ peak corresponds to C-H deformation in lignin and carbohydrates^42^ and it is related with the 4307 cm^−1^ band in the NIR spectrum for it stems from C-H stretching and C-H_2_ deformation combination. To further verify this, we also checked the relationship between the NIR spectra and the mid-IR spectra of *Ganoderma* mycelia based on a two-dimensional correlation spectroscopy of mid-IR and NIR spectra, as shown in Fig. 8. The result unambiguously confirms that the NIR range (5268.8–4000 cm^−1^) is most related to the mid-IR (1422–1376 cm^−1^) range which is mainly ascribed to polysaccharide in different *Ganoderma* stains.

To be noted, however, although quantification of total polysaccharide content is important for quality control of batch consistency, the total polysaccharide content is not necessarily correlated to health effect directly. As mentioned above, the medicinal value of *Ganoderma* mycelium is closely related to its β-glucans. Fortunately, one of the two selected peaks concerned in this work, namely the peak at 1078 cm^−1^ in FTIR (corresponding to NIR signal at 4405 cm^−1^) is just the characteristic for β-glycosides. Therefore, while our NIR quantification model assisted by FTIR spectral analysis is valid for the assessment of total polysaccharide content, it may also be useful for a rough evaluation of β-glucans.

### Comparison of our model with other quantification models

Considering the difference between mycelium and fruiting body of *Ganoderma* genus, it is understandable that our NIR quantification model is different from other previous models which contain broader spectral range. Although our NIR quantification model requires smaller spectral range, it actually gives rise to better prediction performance for providing larger range of polysaccharide values in the assessment.

It seems that it might be better to include bands such as 5787 and 5935 cm^−1^ bands in the quantification model as they also show relatively large correlation coefficients in Fig. 7. To check this, we chose the spectral range 6048–4000 cm^−1^ for comparison. The analysis of quantitative models for polysaccharides based on the NIR spectral range of (6048–4000 cm^−1^) for calibration set and prediction set are shown in Figures S2 and S3. We noticed that it requires higher rank for the assessment (results listed in Table S2). Actually, when we just took the two bands at 4307 and 4405 cm^−1^ in the construction of the quantification model, we obtained R^2^ = 0.8505 and RPR = 2.59 for rank = 6 (Table S2). But if we took the other two bands at 5935 and 5787 cm^−1^ into account, R^2^ became smaller (R^2^ = 0.8227) for rank = 6 (Table S2), with smaller RPD 2.37. So the efficiency or accuracy of the quantification model became worse. The reason may be understood with the following: First, we noticed that for the NIR spectra of *Ganoderma* fruiting body sample with high content of starch and cellulose, although the 4307 and 4405 cm^−1^ bands are prominent in all the three spectra, the 5787 and 5935 cm^−1^ bands are diminished in the spectra of cellulose and starch (Figure S4). Second, if we compare the spectrum of *Ganoderma* mycelium with that of *Ganoderma* fruiting body, we will find their NIR spectra are also really quite different (Figure S5). In *Ganoderma* fruiting body, the NIR signals are so weak for the 4307, 4405, 5787 and 5935 cm^−1^ bands that they are almost invisible in the NIR spectra. Moreover, the spectral shapes are also very different. We explain that such a big difference in spectral features may be due to two factors. First, the compositional structure of mycelium and fruiting body is different. *Ganoderma* fruiting body has a very thick and hard crust consisting of high content of cellulose and lignin, whereas *Ganoderma* mycelium has neither cellulose nor lignin (or the contents of cellulose and lignin are almost negligible in *Ganoderma* mycelium). Second, the polysaccharide content between mycelia and fruiting bodies is also significantly different. As reported by Chen *et al*.^31^, the highest content of polysaccharides is about 8.07%. But in our mycelium samples, the content of polysaccharide is normally higher (up to 11.31%).

In summary, we have established an effective approach to polysaccharide content evaluation for *Ganoderma* mycelium samples, in which we utilized mid-infrared spectroscopy for qualitative analysis and NIR spectroscopy for quantitative assessment. The optimized model contains the region of (5268.8–4000 cm^−1^) and with proper pre-treatment it can give rise to satisfactory prediction performance with the Rank = 6, R^2^ = 0.9779, RMSECV = 0.467, RPD = 6.73 in the calibration set, and RMSEP = 0.603, RPD = 3.13, corr. coeff. = 0.9554 for the prediction set. This work therefore not only achieved an effective approach for establishment of a satisfactory quantification model for polysaccharide assessment in *Ganoderma* mycelia, but also set a good example of practical application of NIR spectroscopy in the assessment of *Ganoderma* polysaccharides in industrial production.

## Methods

### Materials

The *G. lingzhi* strain **5.0026** was purchased from China General Microbiological Culture Collection Center (CGMCC), while some other *Ganoderma* strains were collected in 10 different provinces in China including Anhui, Shandong and Sichuan, etc.

In order to make the range of the experimental samples with polysaccharide contents wide enough, 51 *in vitro* axenic preservation *Ganoderma* strains were purchased, exchanged or isolated from wild fruiting bodies. Among them, 38 stains were *G. lingzhi*, 7 stains were *G.applanatum*, 3 stains were *G.sinense*, 2 stains were *G.resinaceum*, and 1 stain was *G.leucocontextum*. Taking into account of the changes in polysaccharide content of mycelia at different fermentation stages, each strain was cultured for 7 days, 14 days and 21 days, respectively. On the 7th day, most mycelia were in the logarithmic growth phase, and they grew very fast; on the 14th day, most culture flasks were filled with mycelia; on the 21st day, all flasks were full of mycelia, and a few strains started to form its fruiting body. All the 153 mycelium samples were washed with ddH_2_O for three times, placed into petri dishes at −60 °C for 48 hours to freeze for drying (FD-1D-50, Bilon, China). All these strains were randomly selected into calibration (90 samples) and validation sets (63 samples).

All the *Ganoderma* strains were activated in PDA solid medium and then transferred into Potato Dextrose Broth for 7 days, 14 days and 21 days, respectively. For the mid-IR measurements, the mycelia were lyophilized and pulverized into powders for future testing.

Sample grouping and polysaccharide contents (reference and predicated value) were shown in the Table S3. Many of the mycelia were morphologically different in terms of size, color, and viscous degree of the culture solution with some samples demonstrated in Figure S6.

### Extraction and purification of Ganoderma polysaccharides

140 g fermented *Ganoderma* mycelia dried powder, with adding 7 L ddH_2_O, was placed into 70 °C hot water bath for 2 hours for the polysaccharide extraction. The extract liquid was then taken into centrifuge tubes, centrifuged at 4400 rcf/g for 10 min (3K15, Sigma Laborzentrifugen, Germany), kept at 20 °C for 15 min, and then the mycelia precipitate was separated from the crude water-soluble polysaccharide supernatant. The supernatant was then concentrated in a rotary evaporator under reduced pressure at 60 °C to get 850 ml of vacuum-concentrated liquid. The concentrated extract solution was precipitated with 3.4 L ethanol and kept at 4 °C overnight. The precipitate was obtained by centrifugation at 4400 rcf/g for 15 min, and then dried at 45 °C for 2 hours, giving the crude polysaccharides. The crude polysaccharide was then re-dissolved with 800 ml ddH_2_O. 500 ml of the re-dissolved polysaccharide was treated with Sevag reagent (1:4 n-butanol: chloroform, v/v, 120 ml) to remove the proteins inside the solution^39^. The mixture was violently oscillated for 30 min and centrifugated to remove the denatured proteins at the interface between water layer and Sevag reagent layer. The above operation was repeated until no denatured proteins appeared. In order to decolor the solution, 1.5%(v/v) activated charcoal was added to the Sevag-treated crude polysaccharide, with thermostatic water bathing for 40 minutes, then the polysaccharide solution was poured into a dialysis bag, with both ends tightened up, and placed into ddH_2_O. The water was changed every 4 hours, until the color of the dialysate did not change.

### Preparation of freeze-dried polysaccharide samples from Ganoderma genus

Each liquid sample obtained from the steps mentioned above was pipetted and placed into petri dishes at −60 °C for 48 hours to freeze for drying (FD-1D-50, Bilon, China). So we obtained the following samples: a. The GL powder means the drying powder from wet *Ganoderma* culture mycelia. b. The crude GLPS means the extracted crude polysaccharide from the dried powder in 70 °C of hot water. c. The GLPS after condensing is the condensing supernatant using rotary vacuum approach. d. The GLPS after ethanol precipitating means the condensed remnant after ethanol precipitating process. e. The GLPS after Sevag means the polysaccharides removing proteins with the by Sevag method. f. The GLPS after dialysis means the polysaccharide samples which remove small molecular impurity substances after dialyzing operation. These samples were then examined by infrared spectroscopy.

### Measurement of polysaccharides in dried mycelia of different Ganoderma stains

2 ml of 0.012, 0.024, 0.036, 0.048, 0.06, 0.072, and 0.084 mg/ml glucose solutions were prepared, respectively. Then, 6 ml of anthrone reagent was added to each glucose solution and the solution was first kept at room temperature for 15 minutes, and then stored on ice for 15 minutes. When the test tubes were cooled and 3 ml of each sample was read at 625 nm wavelength using UV–vis spectrophotometer (Shimadzu UV-2550, Japan). The spectrum value at 625 nm was recorded for analysis. A standard curve for total carbohydrate assay was generated. The determination coefficient (R^2^) of glucose standard curve is 0.9903, with the standard error less than 0.001^52^.

0.1 g of the lyophilisated sample was mixed with 10 ml ddH_2_O, and placed steady for 1 h. After that, the mixture was placed in 70 °C hot water bath for 2 hours, centrifugated after cooling, and the precipitate was discarded. The supernatant was diluted 20 times and 2 ml sample solution was pipette into a test tube for measurement. 6 ml sulfuric acid solution was added into the test tube and mixed with the sample together. The mixed sample was measured at 625 nm using the UV–Vis spectrophotometer. The content of polysaccharide was then calculated referring to standard curve above (g glucose/100 g sample). And the Ganoderma polysaccharide content was used as reference value for the quantification model^52^.

### Measurements of mid-IR spectra

The samples for mid-IR measurement were prepared by mixing 2 mg of freeze-dried *Ganoderma* mycelia samples with 200 mg of dried potassium bromide followed by pressing under pressure 15 MPa for 3 minutes to make a disk pellet. The samples were then subjected to mid-IR measurements, and the spectral range (4000–400 cm^−1^) was recorded using a Bruker ALPHA-T instrument (Bruker Optics GmbH, Ettlingen, Germany) with a resolution of 4 cm^−1^ and 64 scans per sample. The results were then analyzed using OPUS 7.0 data processing software.

### Measurement of NIR spectra

A FT-NIR spectrometer (NIR MPA, Bruker Optik GmbH, Germany) was used to collect the diffuse reflection spectra, with a resolution of 16 cm^−1^ and 32 scans per sample ranged from 12500–4000 cm^−1^. Each sample was tested several times for the average. These results were then analyzed by OPUS 7.0 data processing software.

### Data analysis

Both NIR and mid-IR spectral data were analyzed using OPUS software (Bruker Optik GmbH, Ettlingen, Germany). Before the spectral data analysis, all the spectra were pre-treated using the procedures of vector normalization and baseline correction. After the spectra were collected, the spectra were exported from OPUS software and imported directly into program IBM SPSS Statistics 19 (SPSS) for cluster analysis, and OriginPro 2016 software (OriginLab Corporation, Northampton, Massachusetts, USA.) for figure graphing.

The data analysis methods including moving window partial least squares (mwPLS), interval partial least squares (iPLS) and correlation coefficient were conducted using iToolbox (programmed by Prof. L. Nørgaard,KVL, Denmark, published on http://www.models.kvl.dk/iToolbox)) on Matlab2012b^®^^53,54^. Both mwPLS and iPLS are the efficient algorithms used to optimize the spectral range for a quantification model: mwPLS builds a series of PLS models in a window that moves over the whole spectral region and then locates useful spectral intervals in terms of the least complexity of PLS models reaching a desired error level^53,54^, while iPLS is an interactive extension to PLS which develops local PLS models on equidistant subintervals of the full-spectrum region and focuses on important spectral regions and removing interferences from other regions^50^.

## Electronic supplementary material

Supplementary information
