# Supplementary material for: Assessment of Polysaccharides from Mycelia of genus Ganoderma by Mid-Infrared and Near-Infrared Spectroscopy
Source: Sci Rep. 2018 Jan 8;8:10. doi: 10.1038/s41598-017-18422-7 (PMC5758644; doi:10.1038/s41598-017-18422-7)
Supplement: Supplementary file 1 — Supplementary information [file 41598_2017_18422_MOESM1_ESM.doc]

**Supplementary Material**

Assessment of Polysaccharides from Mycelia of genus *Ganoderma* by Mid-Infrared and Near-Infrared Spectroscopy

**Yuhan Ma^1,2,3^, Huaqi He^1,2,3^, Jingzhu Wu^4^, Chunyang Wang^1,2^, Kuanglin Chao^5^, Qing Huang^1,2^***

^1^Institute of Technical Biology and Agriculture Engineering, Hefei Institutes of Physical Science, Chinese Academy of Sciences, Hefei 230031, China

^2^ National Synchrotron Radiation Laboratory, School of Life Science, University of Science and Technology of China, Hefei 230026, China

^3^College of Life Science, Anhui Science and Technology University, Fengyang, 233100, China

^4^ School of computer and Information Engineering, Beijing Technology and Business University, Beijing 100048, China

^5^ Environmental Microbial and Food Safety Laboratory, Agricultural Research Service, USDA, Beltsville, MD, 20705, USA

***Corresponding author: Prof. Dr. Qing Huang**

**Email: huangq@ipp.ac.cn**


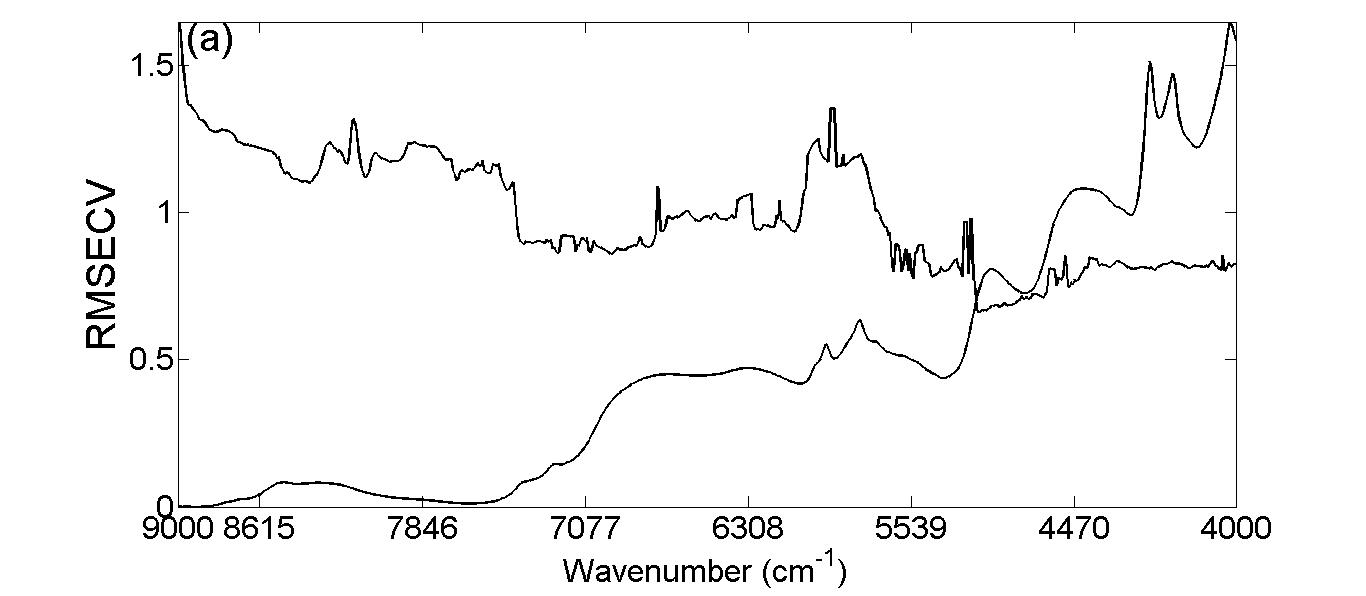


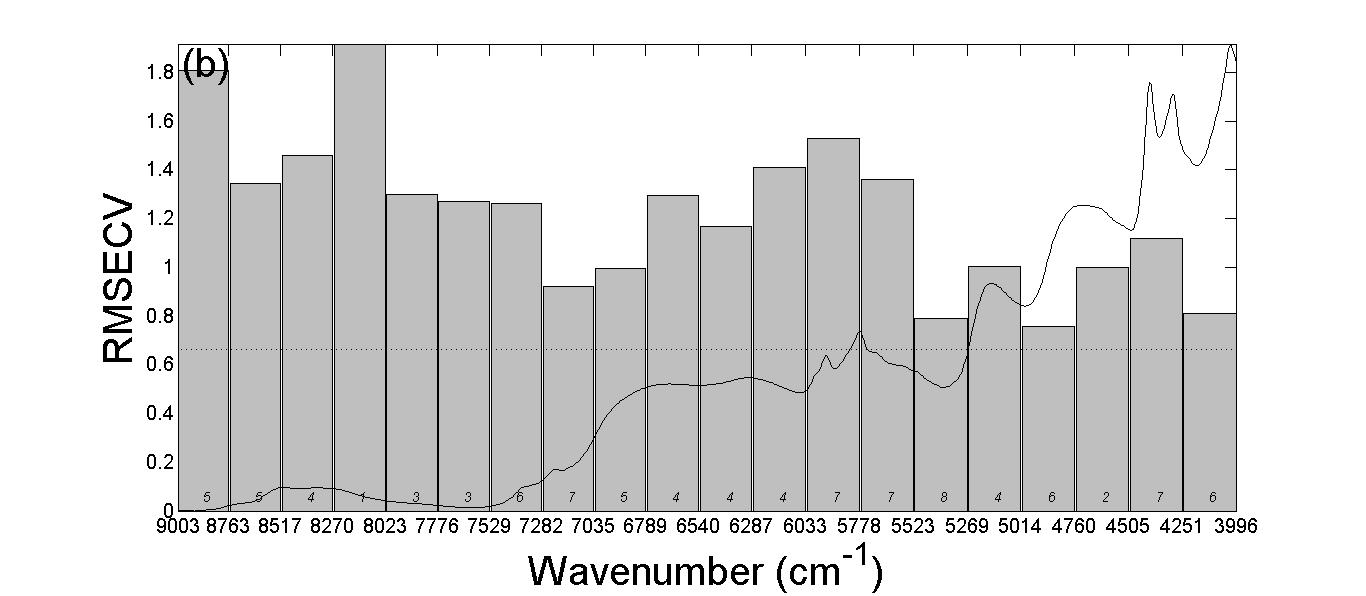


**Figure S1** Analysis of RMSECV based on mwPLS (a) and iPLS (b), confirming that the spectral range (5268.8-4000 cm-1) is best suitable for the NIR quantification model of *Ganoderma* polysaccharides.


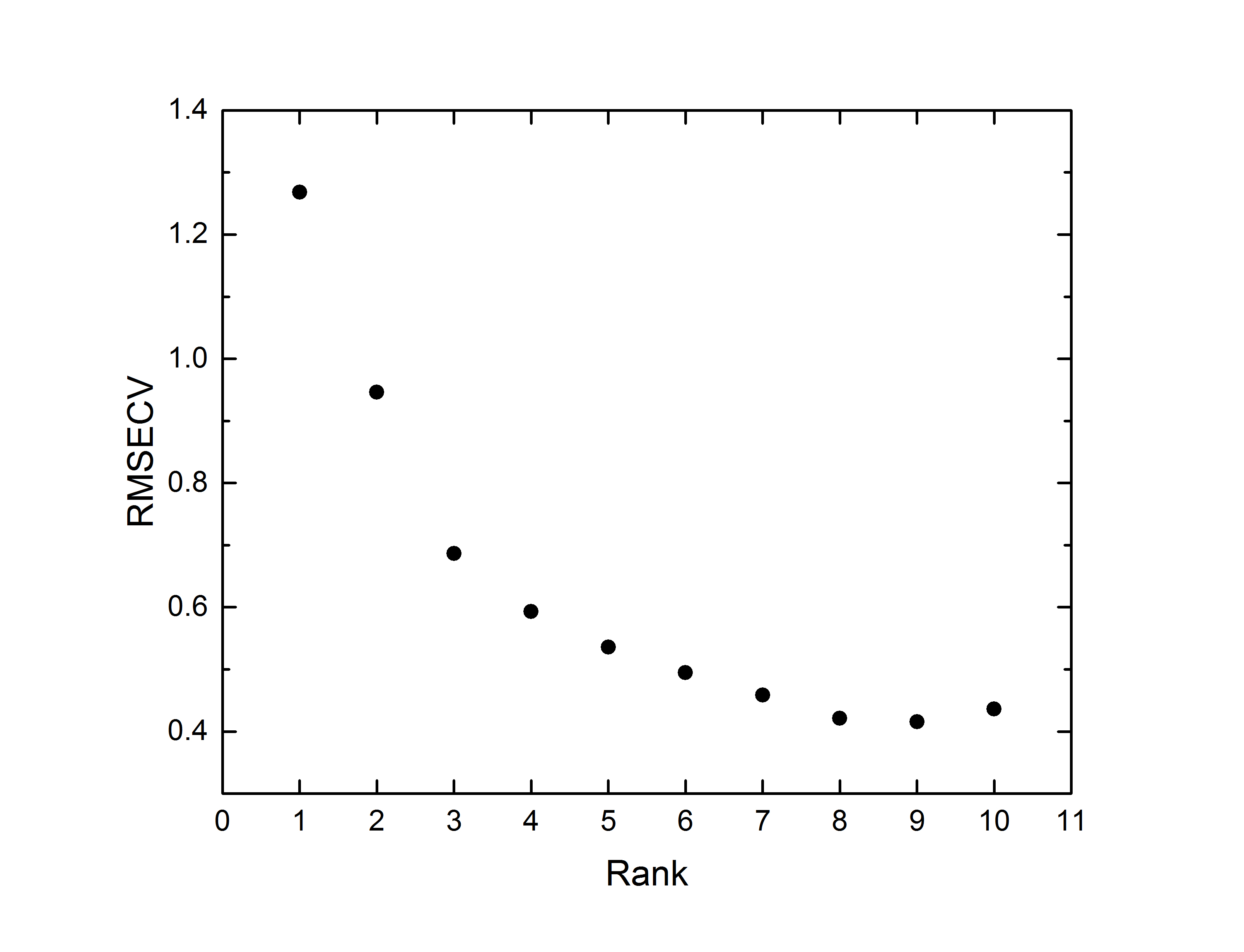

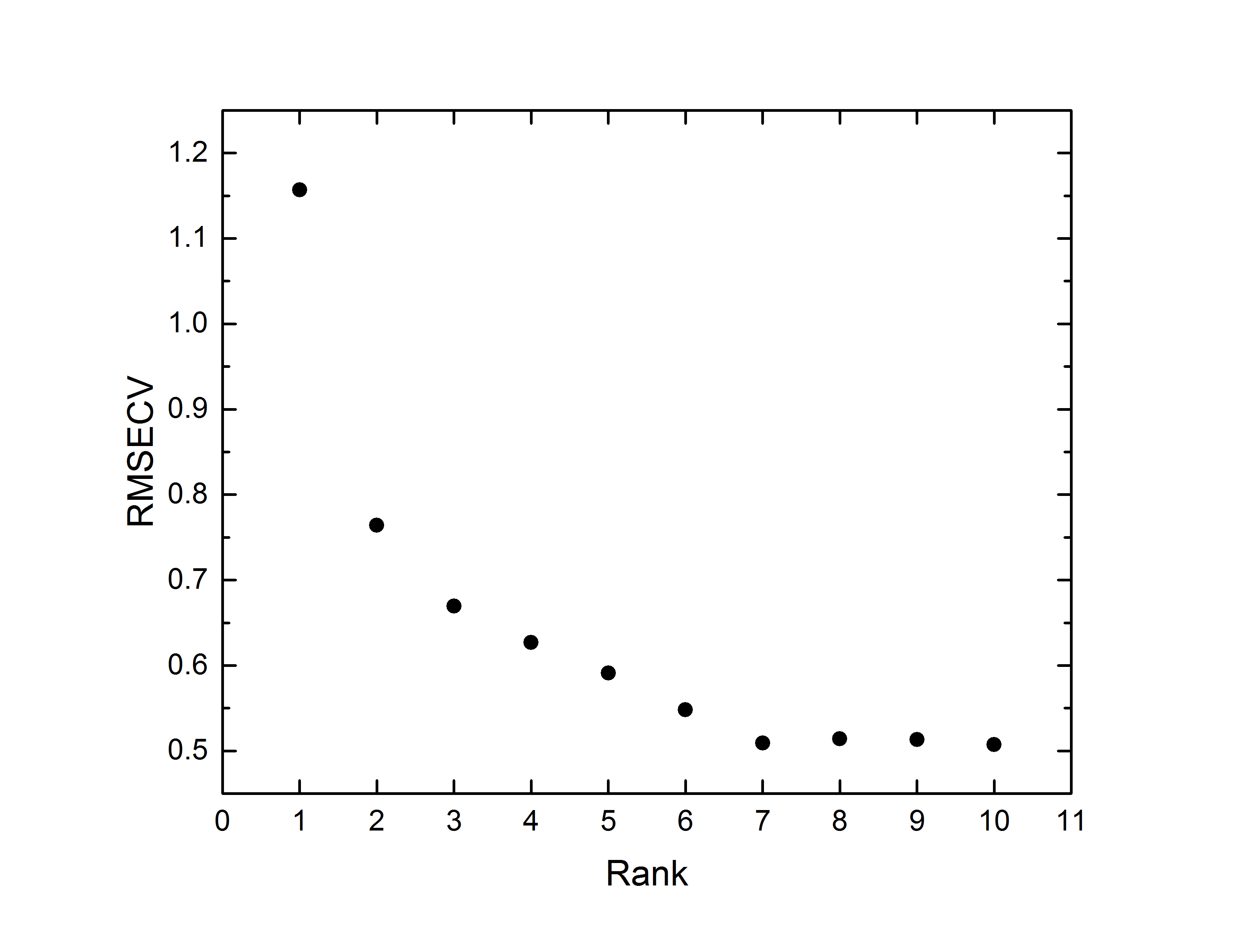


**Figure S2** Plot of RMSECV vs. Rank for the quantitative models of calibration sets for the spectral ranges (a) 6048-4000 cm^-1^; (b) 5268.8-4000 cm^-1^


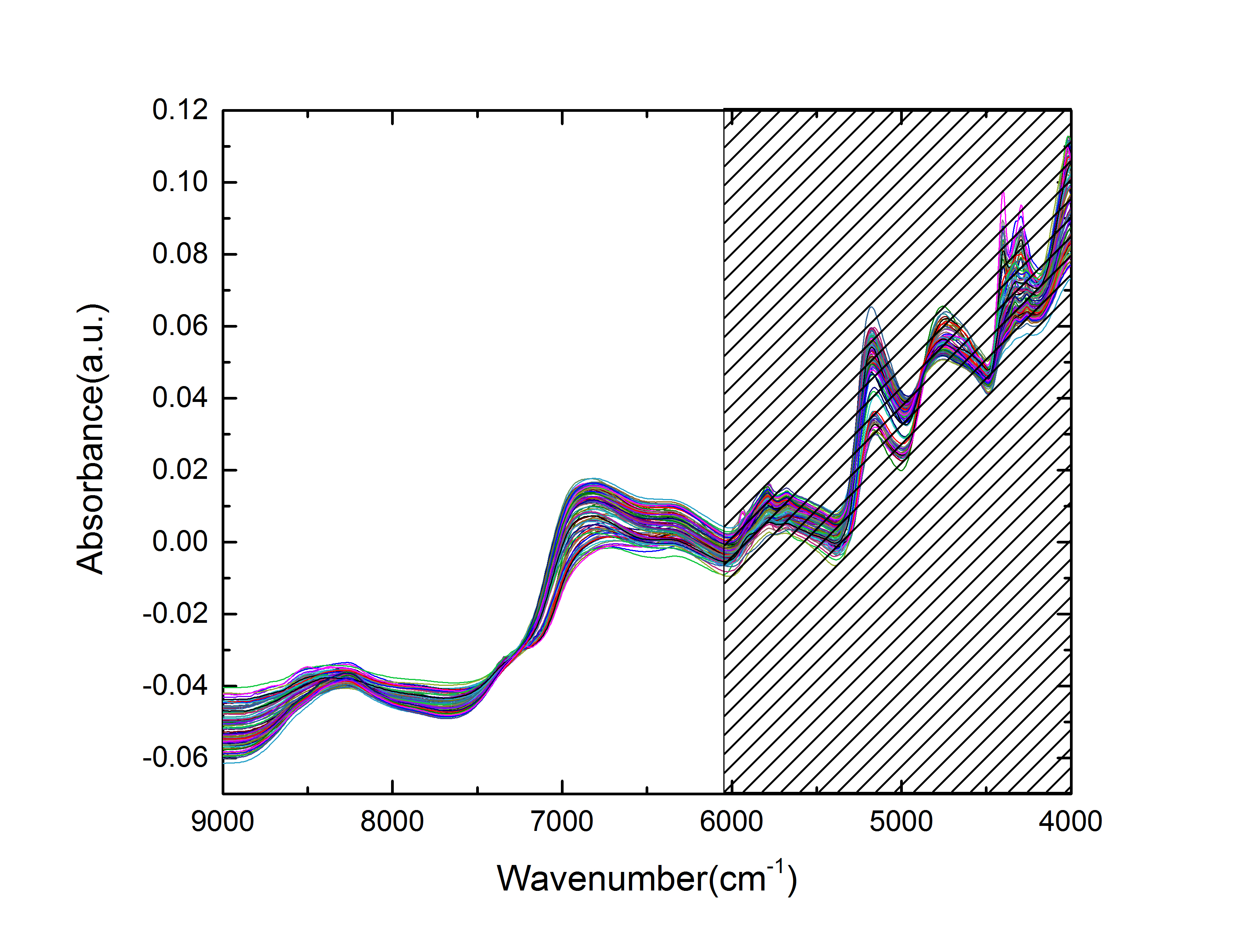

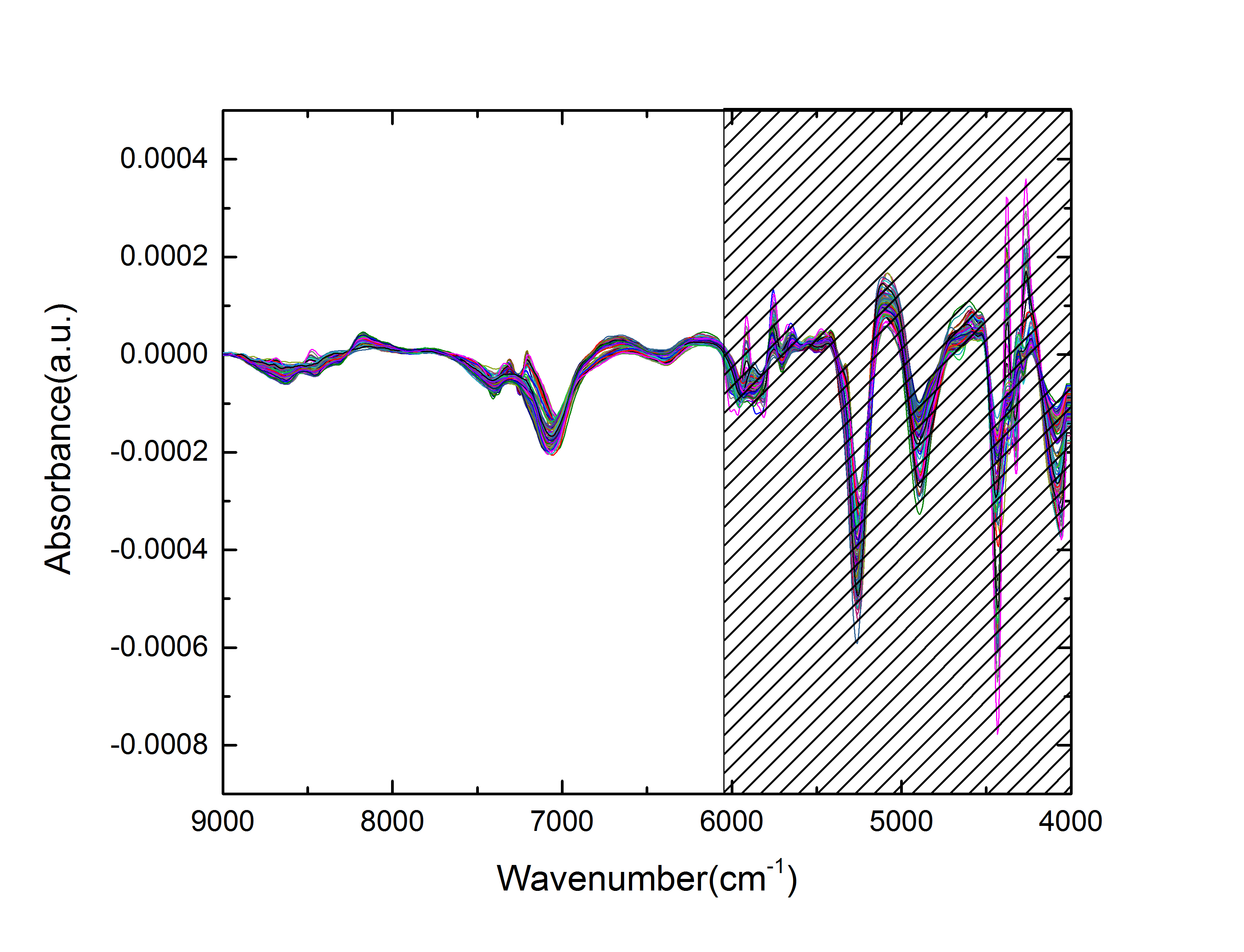


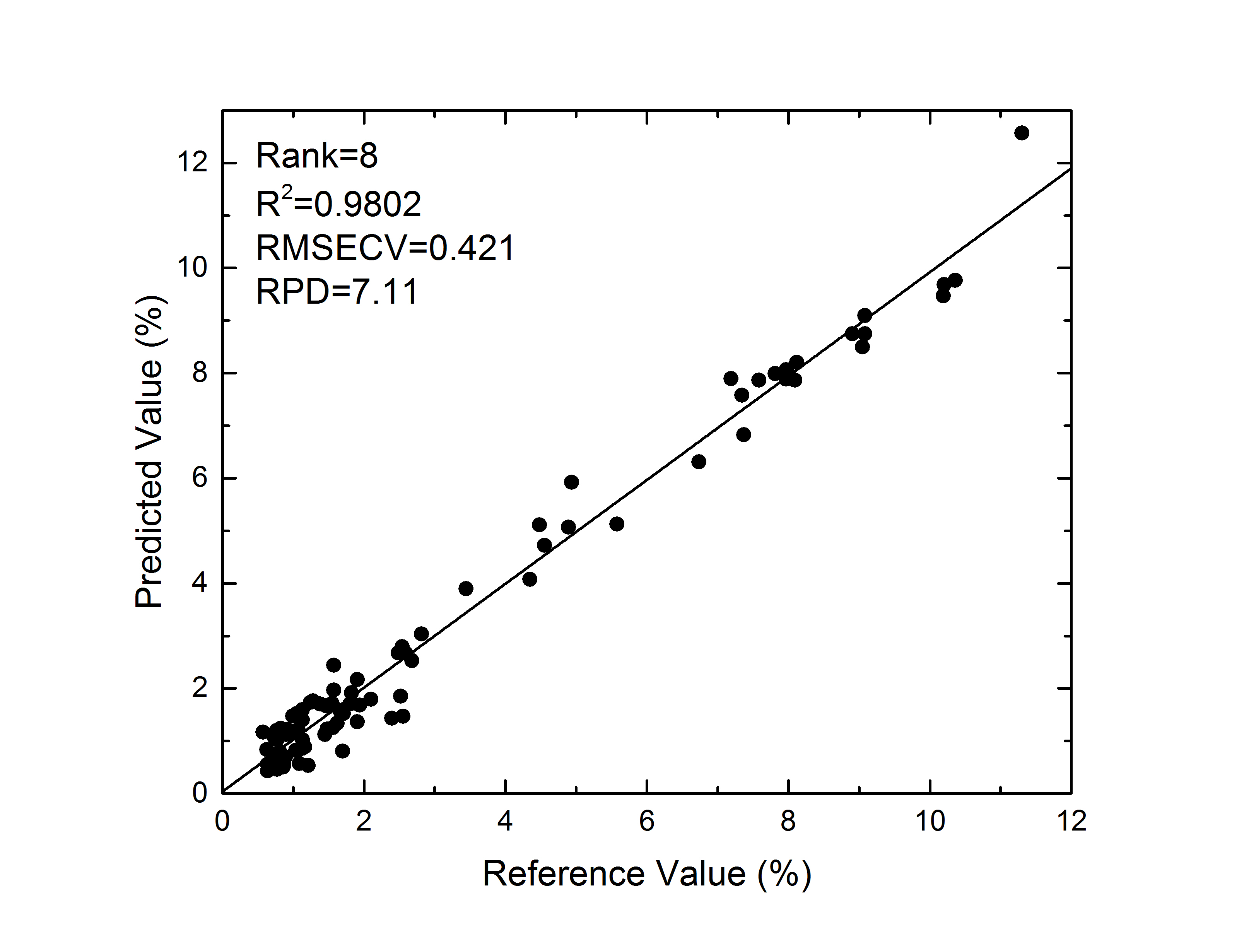

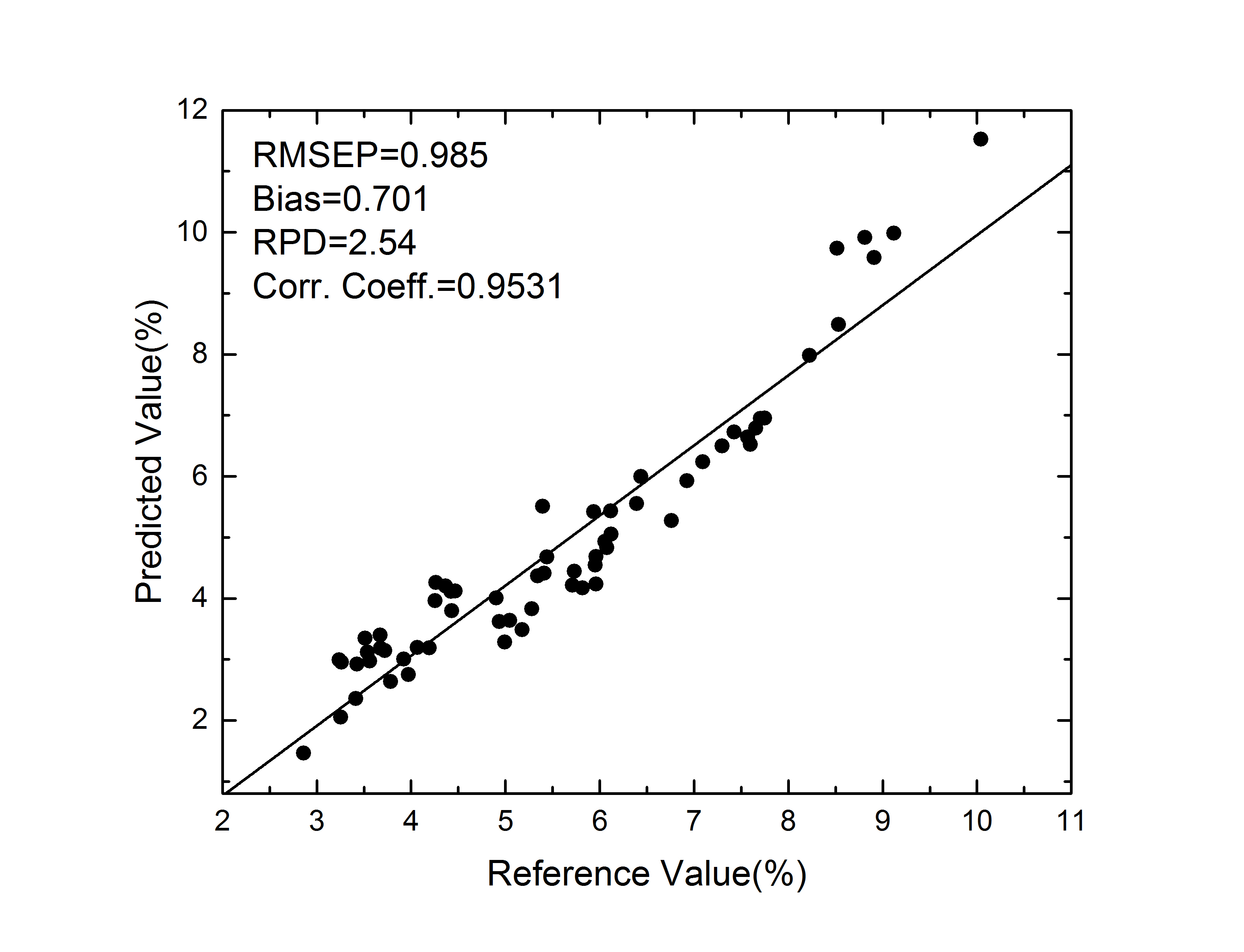


**Figure S3** Top: the original NIR spectra (a) and the first derivative spectra (b) selecting the 6048-4000 cm^-1^ region for the quantification assessment. Bottom: the NIR-based quantitative model for the polysaccharides in the range of (6048-4000 cm^-1^) of calibration set (a) and prediction set (b).


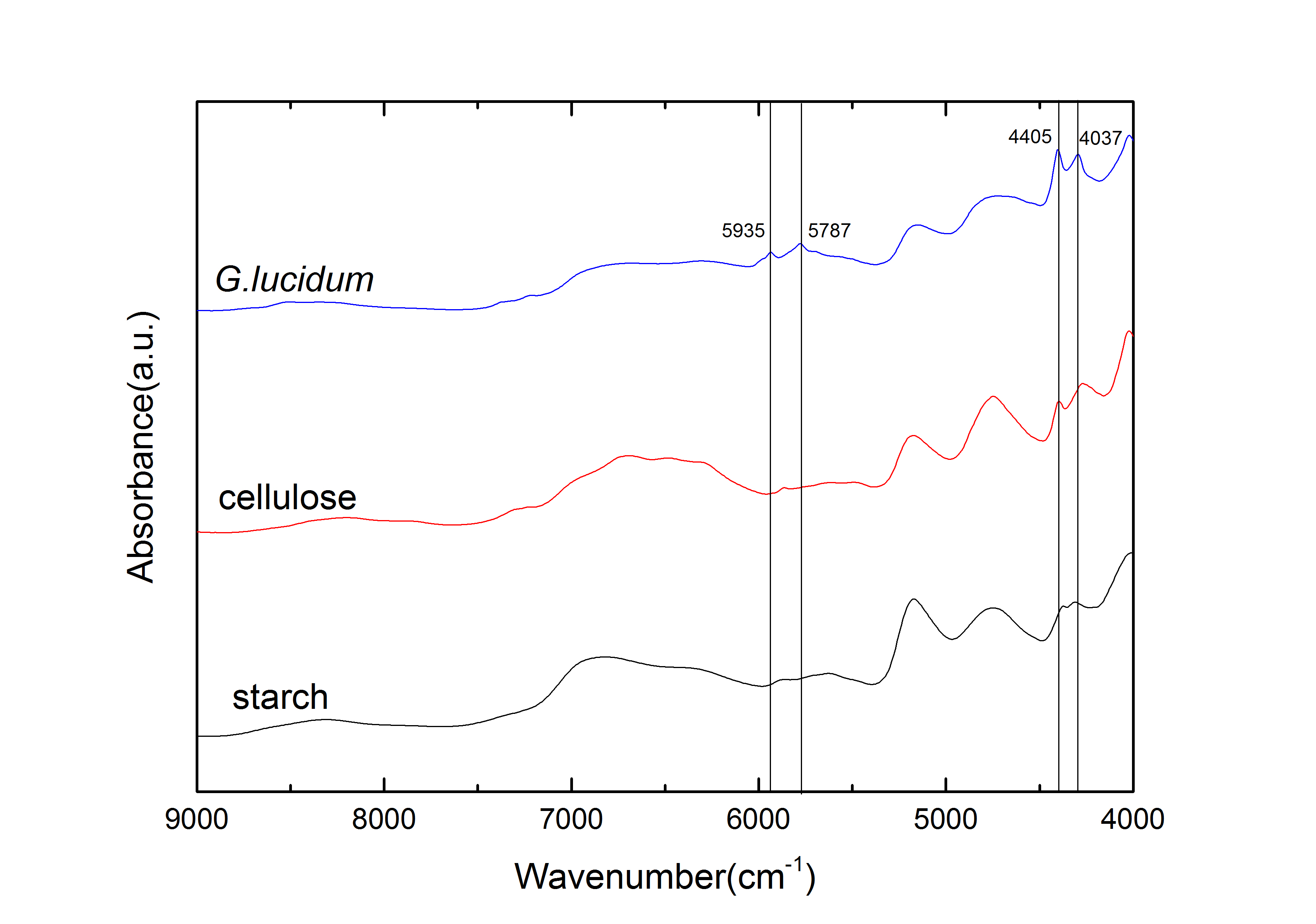


**Figure S4** Comparison of NIR spectra of *G. lingzhi*, cellulose, and starch.


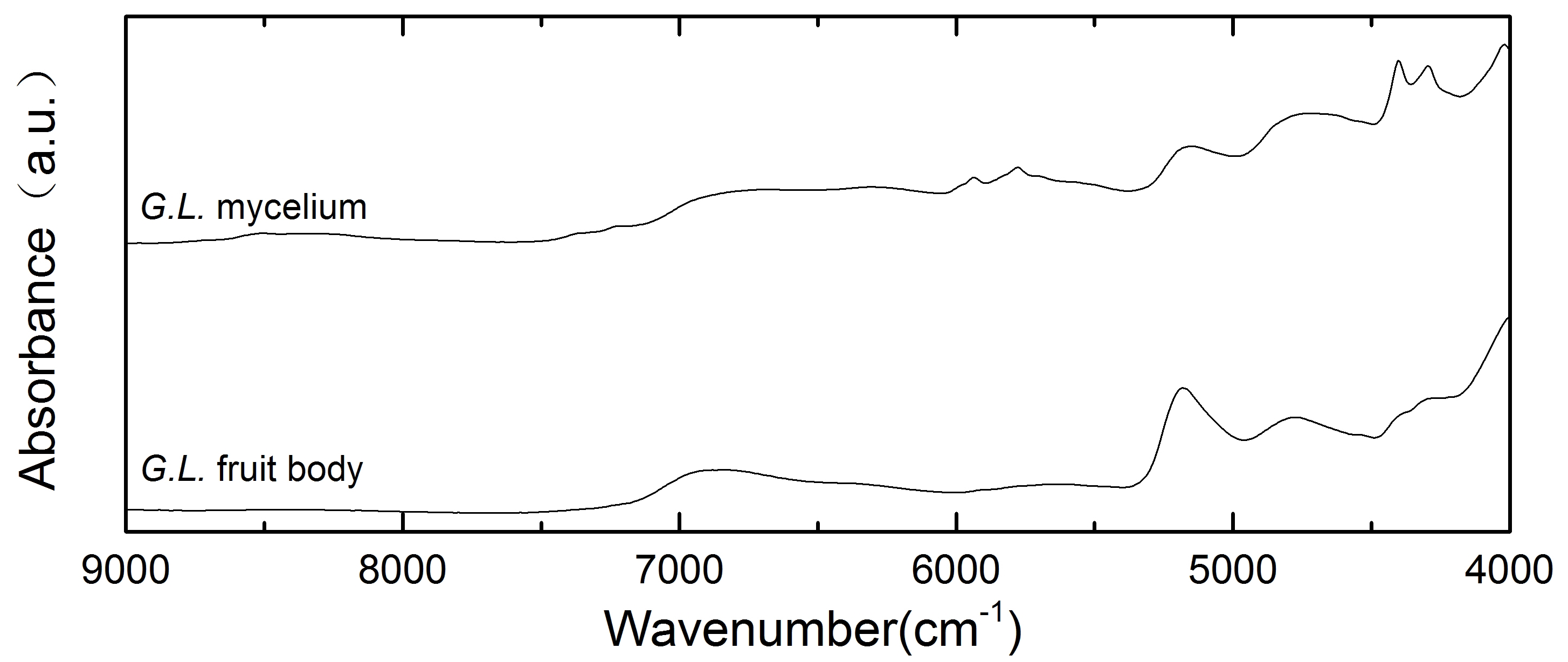


**Figure S5**. Comparison of NIR spectra of *G. lingzhi* between mycelium and fruiting body samples.


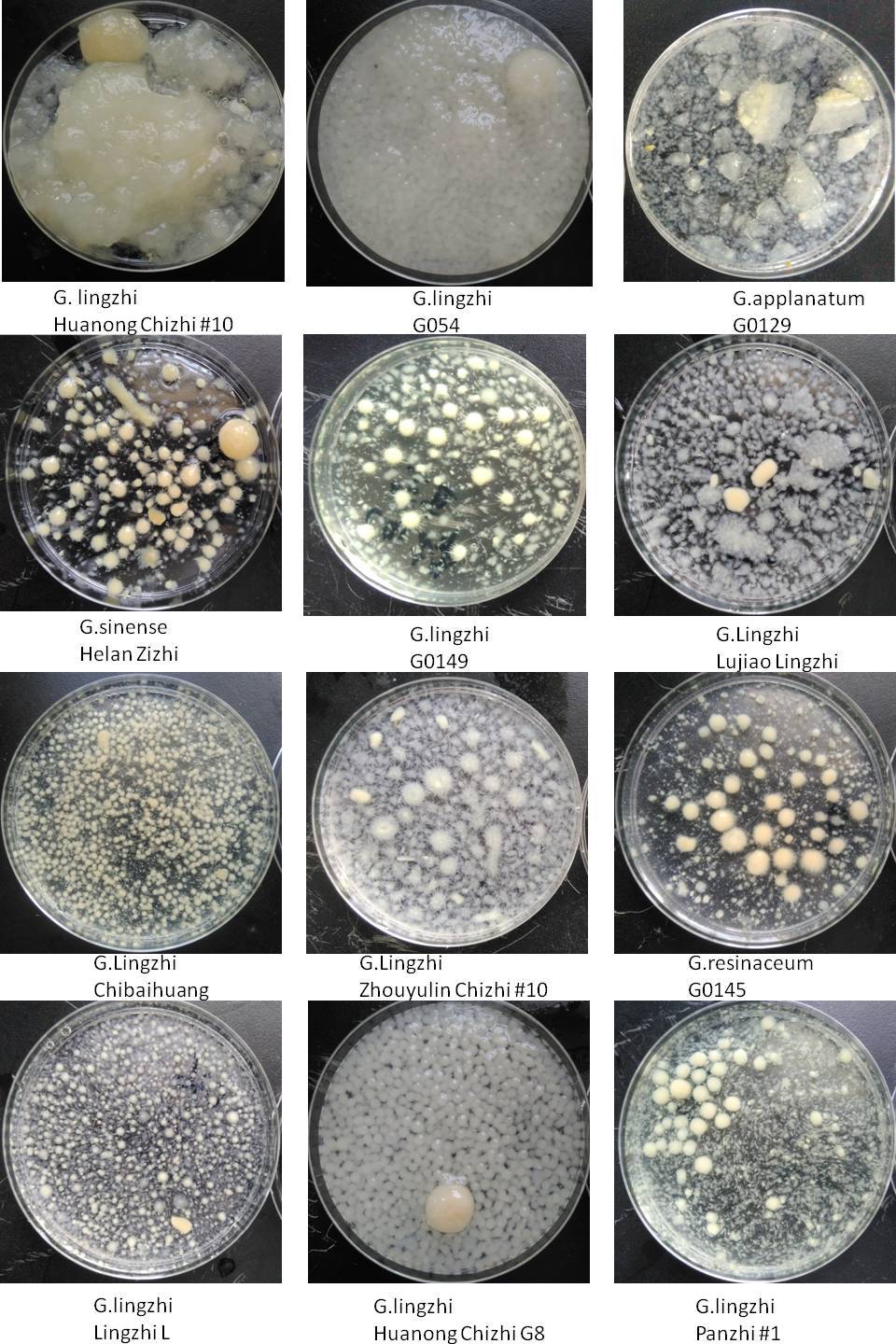


**Figure S6**. The cultured mycelium pellets of different *Ganoderma* strains

**Table S1**. Comparison of performance (rank, R^2^, RMSECV and RPD) of the calibration model depending on pretreatment methods.

| Pre-processing methods | Rank | R^2^ | RMSECV | RPD |
| --- | --- | --- | --- | --- |
| constant offset elimination | 8 | 0.9802 | 0.421 | 7.11 |
| 1st derivative | 7 | 0.9796 | 0.428 | 7 |
| no spectral data preprocessing | 8 | 0.9791 | 0.433 | 6.91 |
| internal standard | 8 | 0.978 | 0.444 | 6.74 |
| 2nd derivative | 9 | 0.9768 | 0.456 | 6.57 |
| straight line subtraction | 7 | 0.9755 | 0.468 | 6.4 |
| min-max normalization | 7 | 0.9706 | 0.513 | 5.84 |
| multiplicative scattering correction | 7 | 0.974 | 0.483 | 6.2 |
| Vector normalization | 7 | 0.9742 | 0.482 | 6.22 |
| 1st derivative + SNV | 9 | 0.9766 | 0.458 | 6.55 |
| 1st derivative + MSC | 8 | 0.9754 | 0.469 | 6.38 |
| 1st derivative + straight line subtraction | 6 | 0.9778 | 0.446 | 6.71 |

(As seen from the results given by the table, the best pre-treatment method is the *constant offset elimination* pre-treatment method for the NIR spectra.)

**Table S2.** Comparison of performance (R^2^, RMSECV and RPD) of PLS calibration model depending on the choice of spectral ranges.

| Calibration regions | Pretreatment methods | Rank | R^2^ | RMSECV | RPD |
| --- | --- | --- | --- | --- | --- |
| 6048-4000 | constant offset elimination | 8 | 0.9802 | 0.421 | 7.11 |
| 6048-4000 | constant offset elimination | 6 | 0.9727 | 0.495 | 6.06 |
| 5268.8-4000 | constant offset elimination | 6 | 0.9779 | 0.467 | 6.73 |
| 4405 & 4307 | constant offset elimination | 5 | 0.8167 | 1.28 | 2.34 |
| 4405 & 4307 | constant offset elimination | 6 | 0.8505 | 1.16 | 2.59 |
| 4307 & 4405 & 5787 & 5935 | constant offset elimination | 6 | 0.8227 | 1.26 | 2.37 |
| 4307 & 4405 & 5787 & 5935 | constant offset elimination | 8 | 0.8436 | 1.18 | 2.53 |
| 7750-4100 | constant offset elimination | 6 | 0.9723 | 0.506 | 6 |
| 7750-4100 | constant offset elimination | 8 | 0.9792 | 0.432 | 6.94 |
| 7750-4100 | 1^st^ derivative + SNV | 8 | 0.9764 | 0.461 | 6.51 |

**Table S3.** *Ganoderma* mycelia samples list for quantification models

Calibration set

| **Number** | **stain Number** | **Strain** | **origin** | **obtain** | **sample name** | **set** | **reference value** | **predicted value** |
| --- | --- | --- | --- | --- | --- | --- | --- | --- |
| 1 | 1 | G.lingzhi 5.0026 | China General Microbiological Culture Collection Center | purchased | 01-7d | calibration | 7.965 | 8.182 |
| 2 | 1 | G.lingzhi 5.0026 | China General Microbiological Culture Collection Center | purchased | 01-21d | calibration | 8.068 | 7.971 |
| 3 | 2 | G.lingzhi WT-1 | Nanjing Agricultural University | purchased | 02-14d | calibration | 6.733 | 5.795 |
| 4 | 3 | G.leucocontextum Bairou | Sichuan Agricultural University | purchased | 03-7d | calibration | 8.121 | 8.006 |
| 5 | 3 | G.leucocontextum Bairou | Sichuan Agricultural University | purchased | 03-14d | calibration | 11.3 | 11.31 |
| 6 | 4 | G.lingzhi Chuanyuanzhi #1 | Sichuan Agricultural University | purchased | 04-7d | calibration | 5.575 | 5.337 |
| 7 | 4 | G.lingzhi Chuanyuanzhi #1 | Sichuan Agricultural University | purchased | 04-14d | calibration | 7.372 | 7.107 |
| 8 | 5 | G.lingzhi Chuanzhi #6 | Sichuan Agricultural University | purchased | 05-7d | calibration | 8.909 | 8.889 |
| 9 | 5 | G.lingzhi Chuanzhi #6 | Sichuan Agricultural University | purchased | 05-14d | calibration | 10.2 | 9.739 |
| 10 | 6 | G.lingzhi Dahuangban | Anhui Agricultural University | purchased | 06-7d | calibration | 9.047 | 8.639 |
| 11 | 6 | G.lingzhi Dahuangban | Anhui Agricultural University | purchased | 06-14d | calibration | 7.583 | 8.182 |
| 12 | 6 | G.lingzhi Dahuangban | Anhui Agricultural University | purchased | 06-21d | calibration | 10.19 | 9.109 |
| 13 | 7 | G.sinense Heizhi | Anhui Agricultural University | purchased | 07-14d | calibration | 9.079 | 8.477 |
| 14 | 7 | G.sinense Heizhi | Anhui Agricultural University | purchased | 07-21d | calibration | 4.481 | 4.763 |
| 15 | 8 | G.lingzhi Hongzhi | Sichuan Agricultural University | purchased | 08-7d | calibration | 4.939 | 5.511 |
| 16 | 8 | G.lingzhi Hongzhi | Sichuan Agricultural University | purchased | 08-21d | calibration | 3.444 | 4.069 |
| 17 | 9 | G.lingzhi Hongzhi #10 | Anhui Agricultural University | purchased | 09-7d | calibration | 7.191 | 8.344 |
| 18 | 9 | G.lingzhi Hongzhi #10 | Anhui Agricultural University | purchased | 09-14d | calibration | 4.89 | 4.605 |
| 19 | 10 | G.lingzhi Panzhi #1 | Sichuan Agricultural University | purchased | 10-14d | calibration | 7.815 | 7.959 |
| 20 | 10 | G.lingzhi Panzhi #1 | Sichuan Agricultural University | purchased | 10-21d | calibration | 4.345 | 4.143 |
| 21 | 12 | G.lingzhi 20120715-8 | Anhui | isolated | 12-14d | calibration | 10.36 | 10.2 |
| 22 | 12 | G.lingzhi 20120715-8 | Anhui | isolated | 12-21d | calibration | 9.081 | 9.266 |
| 23 | 13 | G.applanatum 20120901-5 | Anhui | isolated | 13-21d | calibration | 8.09 | 7.881 |
| 24 | 14 | G.lingzhi 20120901-8 | Anhui | isolated | 14-14d | calibration | 4.552 | 4.447 |
| 25 | 14 | G.lingzhi 20120901-8 | Anhui | isolated | 14-21d | calibration | 7.343 | 8.159 |
| 26 | 15 | G.lingzhi G0126 | shanghaiacademy of agricultural science ; edible fungi research institute | purchased | 15-14d | calibration | 2.395 | 1.619 |
| 27 | 15 | G.lingzhi G0126 | shanghaiacademy of agricultural science ; edible fungi research institute | purchased | 15-21d | calibration | 0.914 | 1.482 |
| 28 | 16 | G.applanatum G0129 | shanghaiacademy of agricultural science ; edible fungi research institute | purchased | 16-14d | calibration | 1.039 | 1.036 |
| 29 | 17 | G.lingzhi G0144 | shanghaiacademy of agricultural science ; edible fungi research institute | purchased | 17-7d | calibration | 1.7 | 0.9 |
| 30 | 17 | G.lingzhi G0144 | shanghaiacademy of agricultural science ; edible fungi research institute | purchased | 17-21d | calibration | 1.085 | 0.539 |
| 31 | 18 | G.resinaceum G0145G.resina | shanghaiacademy of agricultural science ; edible fungi research institute | purchased | 18-21d | calibration | 0.8 | 0.6671 |
| 32 | 19 | G.resinaceum G0149 | shanghaiacademy of agricultural science ; edible fungi research institute | purchased | 19-7d | calibration | 1.62 | 1.267 |
| 33 | 19 | G.resinaceum G0149 | shanghaiacademy of agricultural science ; edible fungi research institute | purchased | 19-21d | calibration | 0.902 | 1.354 |
| 34 | 20 | G.applanatum Bailingzhi 20120721-1 | isolated from wild fruiting body in Jingde, Anhui | isolated | 20-7d | calibration | 1.711 | 1.072 |
| 35 | 21 | G.lingzhi Chibaihuang | Anhui | isolated | 21-7d | calibration | 1.13 | 1.688 |
| 36 | 21 | G.lingzhi Chibaihuang | Anhui | isolated | 21-21d | calibration | 2.817 | 3.309 |
| 37 | 22 | G.sinense Gaoyou Heizhi | Gaoyou, Jiangsu | purchased | 22-7d | calibration | 1.905 | 2.24 |
| 38 | 22 | G.sinense Gaoyou Heizhi | Gaoyou, Jiangsu | purchased | 22-14d | calibration | 1.666 | 1.522 |
| 39 | 23 | G.sinense Helan Zizhi | Gaoyou, Jiangsu | purchased | 23-7d | calibration | 0.891 | 0.5757 |
| 40 | 23 | G.sinense Helan Zizhi | Gaoyou, Jiangsu | purchased | 23-14d | calibration | 0.766 | 1.259 |
| 41 | 24 | G.lingzhi Huannong #10 | Jinzhai, Anhui | purchased | 24-21d | calibration | 2.486 | 2.958 |
| 42 | 25 | G.lingzhi Jinzhai Chizhi | Jinzhai, Anhui | purchased | 25-7d | calibration | 1.21 | 0.7452 |
| 43 | 26 | G.lingzhi Jinzhai Yesheng | Jinzhai, Anhui | purchased | 26-7d | calibration | 2.589 | 2.801 |
| 44 | 26 | G.lingzhi Jinzhai Yesheng | Jinzhai, Anhui | purchased | 26-21d | calibration | 2.555 | 1.553 |
| 45 | 27 | G.lingzhi Lingzhi L | Shandong | purchased | 27-7d | calibration | 1.062 | 1.201 |
| 46 | 27 | G.lingzhi Lingzhi L | Shandong | purchased | 27-21d | calibration | 0.572 | 0.9329 |
| 47 | 28 | G.lingzhi Lujiao Lingzhi | Gaoyou, Jiangsu | purchased | 28-21d | calibration | 1.472 | 1.569 |
| 48 | 29 | G.lingzhi Taishan Lingzhi T | Shandong | purchased | 29-7d | calibration | 0.731 | 0.8434 |
| 49 | 29 | G.lingzhi Taishan Lingzhi T | Shandong | purchased | 29-14d | calibration | 1.13 | 0.466 |
| 50 | 30 | G.lingzhi Yangzhou Lingzhi | Yangzhou, Jiangsu | purchased | 30-7d | calibration | 1.073 | 1.217 |
| 51 | 30 | G.lingzhi Yangzhou Lingzhi | Yangzhou, Jiangsu | purchased | 30-21d | calibration | 1.13 | 0.7961 |
| 52 | 31 | G.applanatum Yunzhi | Jingde, Anhui | isolated | 31-7d | calibration | 0.64 | 0.6226 |
| 53 | 31 | G.applanatum Yunzhi | Jingde, Anhui | isolated | 31-21d | calibration | 1.461 | 1.917 |
| 54 | 32 | G.lingzhi Zhaoqing Lingzhi | Zhaoqing, Guangdong | isolated | 32-7d | calibration | 1.119 | 1.171 |
| 55 | 32 | G.lingzhi Zhaoqing Lingzhi | Zhaoqing, Guangdong | isolated | 32-14d | calibration | 0.64 | 0.5476 |
| 56 | 32 | G.lingzhi Zhaoqing Lingzhi | Zhaoqing, Guangdong | isolated | 32-21d | calibration | 1.164 | 0.5593 |
| 57 | 34 | G.lingzhi G0143 | shanghaiacademy of agricultural science ; edible fungi research institute | purchased | 34-7d | calibration | 0.811 | 0.7867 |
| 58 | 34 | G.lingzhi G0143 | shanghaiacademy of agricultural science ; edible fungi research institute | purchased | 34-21d | calibration | 0.777 | 0.3145 |
| 59 | 35 | G.applanatum 20110824 | Anhui | isolated | 35-14d | calibration | 0.868 | 0.4282 |
| 60 | 36 | G.applanatum 20110901-5 | Anhui | isolated | 36-7d | calibration | 0.948 | 0.9987 |
| 61 | 36 | G.applanatum 20110901-5 | Anhui | isolated | 36-14d | calibration | 0.857 | 0.5413 |
| 62 | 37 | G.applanatum 20120721-4 | Anhui | isolated | 37-7d | calibration | 1.028 | 1.131 |
| 63 | 37 | G.applanatum 20120721-4 | Anhui | isolated | 37-14d | calibration | 1.449 | 1.302 |
| 64 | 37 | G.applanatum 20120721-4 | Anhui | isolated | 37-21d | calibration | 0.777 | 1.177 |
| 65 | 39 | G.lingzhi G027 | Nanjing Agricultural University | isolated | 39-7d | calibration | 0.823 | 1.347 |
| 66 | 39 | G.lingzhi G027 | Nanjing Agricultural University | isolated | 39-14d | calibration | 1.552 | 1.491 |
| 67 | 39 | G.lingzhi G027 | Nanjing Agricultural University | isolated | 39-21d | calibration | 0.823 | 1.115 |
| 68 | 41 | G.lingzhi G043 | Nanjing Agricultural University | isolated | 41-14d | calibration | 0.743 | 0.2597 |
| 69 | 41 | G.lingzhi G043 | Nanjing Agricultural University | isolated | 41-21d | calibration | 0.823 | 0.6943 |
| 70 | 42 | G.lingzhi G054 | Nanjing Agricultural University | isolated | 42-21d | calibration | 0.8 | 0.816 |
| 71 | 43 | G.lingzhi Gl055 | Nanjing Agricultural University | isolated | 43-7d | calibration | 1.563 | 1.264 |
| 72 | 43 | G.lingzhi Gl055 | Nanjing Agricultural University | isolated | 43-14d | calibration | 1.005 | 1.193 |
| 73 | 44 | G.lingzhi Huanong #10 | Huazhong Agricultural University | purchased | 44-7d | calibration | 0.629 | 0.8524 |
| 74 | 44 | G.lingzhi Huanong #10 | Huazhong Agricultural University | purchased | 44-14d | calibration | 1.939 | 1.8 |
| 75 | 44 | G.lingzhi Huanong #10 | Huazhong Agricultural University | purchased | 44-21d | calibration | 1.278 | 1.853 |
| 76 | 45 | G.lingzhi Zhouyulin #10 | Hubei | purchased | 45-7d | calibration | 1.825 | 2.165 |
| 77 | 45 | G.lingzhi Zhouyulin #10 | Hubei | purchased | 45-14d | calibration | 1.051 | 1.61 |
| 78 | 46 | G.lingzhi Huanong G8 | Huazhong Agricultural University | purchased | 46-7d | calibration | 2.52 | 1.993 |
| 79 | 46 | G.lingzhi Huanong G8 | Huazhong Agricultural University | purchased | 46-14d | calibration | 1.483 | 1.245 |
| 80 | 46 | G.lingzhi Huanong G8 | Huazhong Agricultural University | purchased | 46-21d | calibration | 1.803 | 1.876 |
| 81 | 47 | G.lingzhi Feixi | Feixi, Anhui | purchased | 47-7d | calibration | 1.575 | 1.963 |
| 82 | 47 | G.lingzhi Feixi | Feixi, Anhui | purchased | 47-14d | calibration | 1.244 | 1.751 |
| 83 | 47 | G.lingzhi Feixi | Feixi, Anhui | purchased | 47-21d | calibration | 0.994 | 1.268 |
| 84 | 48 | G.lingzhi Funiushan | Funiu mountain, Henan | purchased | 48-7d | calibration | 1.381 | 1.87 |
| 85 | 48 | G.lingzhi Funiushan | Funiu mountain, Henan | purchased | 48-21d | calibration | 2.68 | 2.961 |
| 86 | 49 | G.lingzhi Huludao | Huludao, Liaoning | purchased | 49-14d | calibration | 1.905 | 1.279 |
| 87 | 50 | G.lingzhi suzhou | Suzhou, Anhui | purchased | 50-7d | calibration | 2.099 | 1.826 |
| 88 | 50 | G.lingzhi suzhou | Suzhou, Anhui | purchased | 50-21d | calibration | 1.13 | 1.673 |
| 89 | 51 | G.lingzhi Xuzhou | Xuzhou, Jiangsu | purchased | 51-7d | calibration | 1.575 | 2.469 |
| 90 | 51 | G.lingzhi Xuzhou | Xuzhou, Jiangsu | purchased | 51-21d | calibration | 2.543 | 2.745 |

Predication set

| **Number** | **stain Number** | **Strain** | **origin** | **obtain** | **sample name** | **set** | **reference value** | **predicted value** |
| --- | --- | --- | --- | --- | --- | --- | --- | --- |
| 91 | 1 | G.lingzhi 5.0026 | China General Microbiological Culture Collection Center | purchased | 01-14d | prediction | 3.562 | 4.1947 |
| 92 | 2 | G.lingzhi WT-1 | Nanjing Agricultural University | purchased | 02-7d | prediction | 3.254 | 3.3907 |
| 93 | 2 | G.lingzhi WT-1 | Nanjing Agricultural University | purchased | 02-21d | prediction | 3.972 | 3.7714 |
| 94 | 3 | G.leucocontextum Bairou | Sichuan Agricultural University | purchased | 03-21d | prediction | 4.256 | 5.172 |
| 95 | 4 | G.lingzhi Chuanyuanzhi #1 | Sichuan Agricultural University | purchased | 04-21d | prediction | 3.678 | 4.2184 |
| 96 | 5 | G.lingzhi Chuanzhi #6 | Sichuan Agricultural University | purchased | 05-21d | prediction | 4.366 | 5.2164 |
| 97 | 7 | G.sinense Heizhi | Anhui Agricultural University | purchased | 07-7d | prediction | 3.536 | 4.5371 |
| 98 | 8 | G.lingzhi Hongzhi | Sichuan Agricultural University | purchased | 08-14d | prediction | 2.86 | 3.0407 |
| 99 | 9 | G.lingzhi Hongzhi #10 | Anhui Agricultural University | purchased | 09-21d | prediction | 3.236 | 4.2921 |
| 100 | 10 | G.lingzhi Panzhi #1 | Sichuan Agricultural University | purchased | 10-7d | prediction | 3.426 | 4.1377 |
| 101 | 11 | G.lingzhi Qingzhi | Sichuan Agricultural University | purchased | 11-7d | prediction | 3.264 | 4.0794 |
| 102 | 11 | G.lingzhi Qingzhi | Sichuan Agricultural University | purchased | 11-14d | prediction | 3.412 | 3.8064 |
| 103 | 11 | G.lingzhi Qingzhi | Sichuan Agricultural University | purchased | 11-21d | prediction | 3.924 | 4.1523 |
| 104 | 12 | G.lingzhi 20120715-8 | Anhui | isolated | 12-7d | prediction | 4.424 | 5.3887 |
| 105 | 13 | G.applanatum 20120901-5 | Anhui | isolated | 13-7d | prediction | 4.066 | 4.2264 |
| 106 | 13 | G.applanatum 20120901-5 | Anhui | isolated | 13-14d | prediction | 5.44 | 5.8318 |
| 107 | 14 | G.lingzhi 20120901-8 | Anhui | isolated | 14-7d | prediction | 3.722 | 4.1367 |
| 108 | 15 | G.lingzhi G0126 | shanghaiacademy of agricultural science ; edible fungi research institute | purchased | 15-7d | prediction | 3.672 | 4.6938 |
| 109 | 16 | G.applanatum G0129 | shanghaiacademy of agricultural science ; edible fungi research institute | purchased | 16-7d | prediction | 4.192 | 4.3541 |
| 110 | 16 | G.applanatum G0129 | shanghaiacademy of agricultural science ; edible fungi research institute | purchased | 16-21d | prediction | 4.43 | 5.0349 |
| 111 | 17 | G.lingzhi G0144 | shanghaiacademy of agricultural science ; edible fungi research institute | purchased | 17-14d | prediction | 3.514 | 4.4793 |
| 112 | 18 | G.resinaceum G0145G.resina | shanghaiacademy of agricultural science ; edible fungi research institute | purchased | 18-7d | prediction | 4.468 | 5.1605 |
| 113 | 18 | G.resinaceum G0145G.resina | shanghaiacademy of agricultural science ; edible fungi research institute | purchased | 18-14d | prediction | 4.264 | 5.4613 |
| 114 | 19 | G.resinaceum G0149 | shanghaiacademy of agricultural science ; edible fungi research institute | purchased | 19-14d | prediction | 3.782 | 4.0119 |
| 115 | 20 | G.applanatum Bailingzhi 20120721-1 | isolated from wild fruiting body in Jingde, Anhui | isolated | 20-14d | prediction | 5.936 | 6.3153 |
| 116 | 20 | G.applanatum Bailingzhi 20120721-1 | isolated from wild fruiting body in Jingde, Anhui | isolated | 20-21d | prediction | 6.12 | 6.223 |
| 117 | 21 | G.lingzhi Chibaihuang | Anhui | isolated | 21-14d | prediction | 6.116 | 6.4397 |
| 118 | 22 | G.sinense Gaoyou Heizhi | Gaoyou, Jiangsu | purchased | 22-21d | prediction | 6.762 | 6.2459 |
| 119 | 23 | G.sinense Helan Zizhi | Gaoyou, Jiangsu | purchased | 23-21d | prediction | 6.392 | 6.7751 |
| 120 | 24 | G.lingzhi Huannong #10 | Jinzhai, Anhui | purchased | 24-7d | prediction | 5.394 | 6.155 |
| 121 | 24 | G.lingzhi Huannong #10 | Jinzhai, Anhui | purchased | 24-14d | prediction | 5.954 | 5.4817 |
| 122 | 25 | G.lingzhi Jinzhai Chizhi | Jinzhai, Anhui | purchased | 25-14d | prediction | 5.596 | 4.9265 |
| 123 | 25 | G.lingzhi Jinzhai Chizhi | Jinzhai, Anhui | purchased | 25-21d | prediction | 5.962 | 5.4621 |
| 124 | 26 | G.lingzhi Jinzhai Yesheng | Jinzhai, Anhui | purchased | 26-14d | prediction | 6.438 | 6.7053 |
| 125 | 27 | G.lingzhi Lingzhi L | Shandong | purchased | 27-14d | prediction | 5.818 | 5.489 |
| 126 | 28 | G.lingzhi Lujiao Lingzhi | Gaoyou, Jiangsu | purchased | 28-7d | prediction | 6.076 | 6.0239 |
| 127 | 28 | G.lingzhi Lujiao Lingzhi | Gaoyou, Jiangsu | purchased | 28-14d | prediction | 5.28 | 5.0846 |
| 128 | 29 | G.lingzhi Taishan Lingzhi T | Shandong | purchased | 29-21d | prediction | 5.41 | 5.4888 |
| 129 | 30 | G.lingzhi Yangzhou Lingzhi | Yangzhou, Jiangsu | purchased | 30-14d | prediction | 5.178 | 4.5351 |
| 130 | 31 | G.applanatum Yunzhi | Jingde, Anhui | isolated | 31-14d | prediction | 5.342 | 5.6391 |
| 131 | 33 | G.lingzhi G0133 | shanghaiacademy of agricultural science ; edible fungi research institute | purchased | 33-7d | prediction | 6.056 | 6.1117 |
| 132 | 33 | G.lingzhi G0133 | shanghaiacademy of agricultural science ; edible fungi research institute | purchased | 33-14d | prediction | 5.71 | 5.3999 |
| 133 | 33 | G.lingzhi G0133 | shanghaiacademy of agricultural science ; edible fungi research institute | purchased | 33-21d | prediction | 4.904 | 5.0332 |
| 134 | 34 | G.lingzhi G0143 | shanghaiacademy of agricultural science ; edible fungi research institute | purchased | 34-14d | prediction | 4.934 | 4.7156 |
| 135 | 35 | G.applanatum 20110824 | Anhui | isolated | 35-7d | prediction | 7.092 | 7.6185 |
| 136 | 35 | G.applanatum 20110824 | Anhui | isolated | 35-21d | prediction | 7.654 | 8.1772 |
| 137 | 36 | G.applanatum 20110901-5 | Anhui | isolated | 36-21d | prediction | 8.91 | 9.785 |
| 138 | 38 | G.lingzhi G018 | Nanjing Agricultural University | isolated | 38-7d | prediction | 4.098 | 4.2213 |
| 139 | 38 | G.lingzhi G018 | Nanjing Agricultural University | isolated | 38-14d | prediction | 5.046 | 4.7356 |
| 140 | 38 | G.lingzhi G018 | Nanjing Agricultural University | isolated | 38-21d | prediction | 7.568 | 7.8584 |
| 141 | 40 | G.lingzhi G039 | Nanjing Agricultural University | isolated | 40-7d | prediction | 7.596 | 7.7339 |
| 142 | 40 | G.lingzhi G039 | Nanjing Agricultural University | isolated | 40-14d | prediction | 9.118 | 10.334 |
| 143 | 40 | G.lingzhi G039 | Nanjing Agricultural University | isolated | 40-21d | prediction | 7.424 | 7.8385 |
| 144 | 41 | G.lingzhi G043 | Nanjing Agricultural University | isolated | 41-7d | prediction | 5.96 | 5.609 |
| 145 | 42 | G.lingzhi G054 | Nanjing Agricultural University | isolated | 42-7d | prediction | 8.516 | 9.9292 |
| 146 | 42 | G.lingzhi G054 | Nanjing Agricultural University | isolated | 42-14d | prediction | 7.702 | 8.0338 |
| 147 | 43 | G.lingzhi Gl055 | Nanjing Agricultural University | isolated | 43-21d | prediction | 8.226 | 9.0055 |
| 148 | 45 | G.lingzhi Zhouyulin #10 | Hubei | purchased | 45-21d | prediction | 6.924 | 6.9624 |
| 149 | 48 | G.lingzhi Funiushan | Funiu mountain, Henan | purchased | 48-14d | prediction | 7.296 | 7.5128 |
| 150 | 49 | G.lingzhi Huludao | Huludao, Liaoning | purchased | 49-7d | prediction | 7.75 | 7.9906 |
| 151 | 49 | G.lingzhi Huludao | Huludao, Liaoning | purchased | 49-21d | prediction | 9.703 | 10.485 |
| 152 | 50 | G.lingzhi suzhou | Suzhou, Anhui | purchased | 50-14d | prediction | 8.532 | 9.7053 |
| 153 | 51 | G.lingzhi Xuzhou | Xuzhou, Jiangsu | purchased | 51-14d | prediction | 5.732 | 5.5271 |
